# Environmental conditions driven method for automobile cabin pre-conditioning with multi-satisfaction objectives

**DOI:** 10.1371/journal.pone.0266672

**Published:** 2022-05-23

**Authors:** Weijian Li, Jiqing Chen, Fengchong Lan

**Affiliations:** 1 School of Mechanical & Automotive Engineering, South China University of Technology, Guangzhou, GuangDong, China; 2 Guangdong Key Laboratory of Automotive Engineering, Guangzhou, China; Tongji University, CHINA

## Abstract

The optimal initial pre-conditioning parameter is essential to properly adjust the temperature within the cabin in an effective and accurate way, especially while passengers’ thermal comfort and energy-saving properties are both considered. Under the various environmental thermal loads, the pre-conditioning solutions resulting from those pre-fixed cooling parameters are unfeasible for achieving accurately passengers’ comfort temperature. In addition, it is also difficult in such a narrow car space to identify a lot of local attributes due to the different material properties and sizes of a variety of structural parts that have various thermal responses to environmental conditions. This paper presents a data-driven decision model to numerically identify the degrees of the cabin thermal characteristic to determine satisfactory pre-conditioning parameter schemes. Initially, based on the thermal data within a vehicle recorded through the whole year at a selected hot climate region of the Middle East, the study levels multiple climate scenes corresponding to change in the cabin air temperature. Then three classification algorithms (Support Vector Machines, Decision Tree, and K-nearest neighbor model) are used to comparatively identify climate levels according to the input conditions. Based on the identified climate level, an appropriate parameters scheme for this level is applied. A comprehensive evaluation index (*CEI*) is proposed to characterize the passengers’ satisfaction in numerical computation, on considering multi-satisfaction objectives including Predicted Mean Vote (PMV), local temperature, air quality, and energy efficiency; and it formulates the pre-conditioning parameter scheme for each climate scene with *CEI*. Several scene cases are carried out to verify the effectiveness of the proposed models. The result shows that the pre-conditioning schemes of the model can effectively satisfy passengers in multi-satisfaction objectives.

## Introduction

Intelligent and automatic systems in vehicles have been receiving attention for recent years. In such an autonomous vehicle, the HVAC (Heating, Ventilation, and Air Conditioning) system is required to enable self-serving passengers’ thermal comfort with energy-saving strategies. The thermal state of the cabin is the result of its interaction with the external environment, such as solar radiation, air as well as dust [1–3], and fails to meet human comfort objectives [4, 5]. Without the cabin pre-conditioning, passengers entering the cabin have to endure long periods of thermal discomfort and intense self-regulation, as well as toxic volatiles [6, 7]. The HVAC is also bound to take time to satisfy comfort objectives with additional energy. How to achieve efficient and accurate HVAC pre-conditioning controlling according to the different initial thermal states in the cabin is a problem to be solved. Actually, city layout on landscapes planning makes local climates strongly different between road and outdoor semi-open spaces, where parked cars experience various thermal states in cabins, although it is the same entire climate at one area district. As known in statistics, the ratio of the parking time is about 10–20 to the driving during the automobile serving period [8], while more than one-third are open-air parked [9]. Although HVAC systems of the building already apply pre-conditioning, the vehicle cabin has a more changeable thermal environment and complex interactions than buildings [10]. In such a narrow car space, the different material properties and sizes of structural parts have various thermal responses to environmental conditions. An inappropriate value might be set without a correct perception of the cabin environment [11], resulting in dissatisfaction and energy waste [12, 13]. Therefore, cabin pre-conditioning strategies must consider the dynamically changing thermal states in cabins.

Many studies have been carried out to improve the thermal environment in vehicles [14, 15]. Healthy ventilation and temperature control strategies are applied to reduce the threat of harmful gases and viruses in a vehicle cabin [15]. Supply control of variable air volumes helps to meet different thermal demand objectives in cabin [16]. Thermal condition strategies based on thermal loads and thermal comfort can effectively reduce the energy consumption of electric vehicles [17–19]. Related strategies focus on thermal comfort and energy management when the vehicle environment stabilizes, while effective management strategies for the initial thermal environment is lacking. For cabin pre-conditioning, serval studies applied additional structural to reduce the heat accumulated by parked vehicles [20–24]. Pan et al. [20] and Lee et al. [22] by adding a pre-ventilation system, were to reduce cabin thermal load with fan and solar sunroof. Maan et al. [24] proposed a device to cool the vehicle cabin with compressed air by vaporizing endothermic, but the safety of the air tank was another problem to be worth considering. Related studies about cabin pre-conditioning with fixed cooling parameters cannot adapt to the dynamically changing urban climate, which would provide an unsatisfactory outcome once the environmental conditions deviate from the scope of their setting conditions. As reviewed, a systematic understanding of urban climate on vehicle cabins and perfect cooling strategies are lacking. It is necessary to for pre-conditioning identify the cabin thermal environment characteristics formed by climate conditions.

Evaluating passengers’ satisfaction with thermal comfort is another issue for the cabin pre-conditioning. In the narrow cabin, the thermal states and seat surface contact with the passenger both directly affect the passenger’s thermal sensation. The impact of tactility factors cannot be ignored to human comfort. An effective cabin pre-conditioning should meet the human thermal needs for these factors, especially the surface temperature, air quality, and energy consumption. The PMV- PPD (Predicted Mean Vote- Predicted Percentage Dissatisfied) is most commonly used for assessing passenger satisfaction with air thermal state [25, 26], but the evaluation methods for other factors are still needed. How to evaluate the passenger’s satisfaction with the cabin pre-conditioning comprehensively troubles the planning of the strategy.

A suitable pre-conditioning decision method is one of the subjects for the intelligent vehicles adapting to changing external environment conditions. Reliable data on cabin thermal characteristics is essential to pre-conditioning decisions. The data in the study come from experiments conducted in a tropical desert climate which collect the thermal properties outside and inside the cabin. The relationship of thermal responses between the inside and outside cabin was analyzed by thermal transfer. According to cabin air temperature varied from hot to neutral, the cabin thermal environmental variable would be divided into multiple levels corresponding to the cooling amplitude in pre-conditioning. A data-driven decision model with Cubic Support Vector Machines is proposed to identify the cabin thermal environment level and determine pre-conditioning schemes. Considering the multiple satisfaction objectives, a comprehensive evaluation index *CEI* is suggested to evaluate the passengers’ satisfaction on both thermal comfort, seat temperature, air quality, and energy consumption. *CEI* effectively provides assessments for pre-conditioning parameter schemes.

## Materials and methods

### Cabin pre-conditioning strategy based on thermal environment levels

**Fig 1:**
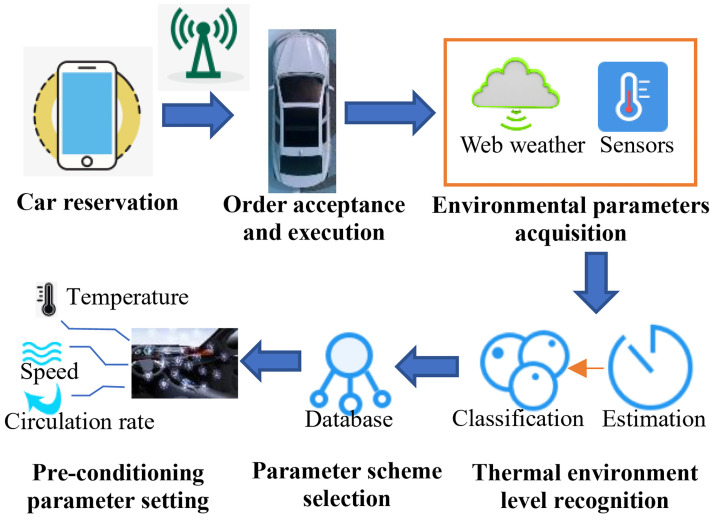
Implementation process of the vehicle HVAC pre-conditioning system. The system was designed for internet-enabled vehicles; therefore, passengers send network commands to activate parked vehicle systems for ventilation. The pre-conditioning system runs 10 minutes before passenger demand. The vehicle system specifies the optimal air conditioning parameters based on the current environmental conditions to the satisfy the passengers.

Driverless technology and intelligent vehicles place new demands on the management of the thermal environment in the vehicle cabin. When passengers need to use parked vehicles, pre-conditioning of the HVAC is asked to eliminate the worse environment in the cabin formed during the parking process. Among these, the cabin pre-conditioning with HVAC means that HVAC cools or heats the cabin environment before the passenger enters with energy-saving, thermal comfort and efficiency, activating in advance via network reservation commands. Fig 1 shows an outline of the HVAC pre-conditioning implementation with environment data driven. The pre-conditioning system obtains environmental parameters and predicts component temperatures firstly. Then, the level of the thermal environment state is identified based on measured parameters, and the corresponding level of pre-conditioning solution in the database is called up to set the cooling parameters of the HVAC. The implementation of multi-satisfaction settings parameters for pre-conditioning in different scenarios is the main focus of this research.

**Fig 2:**
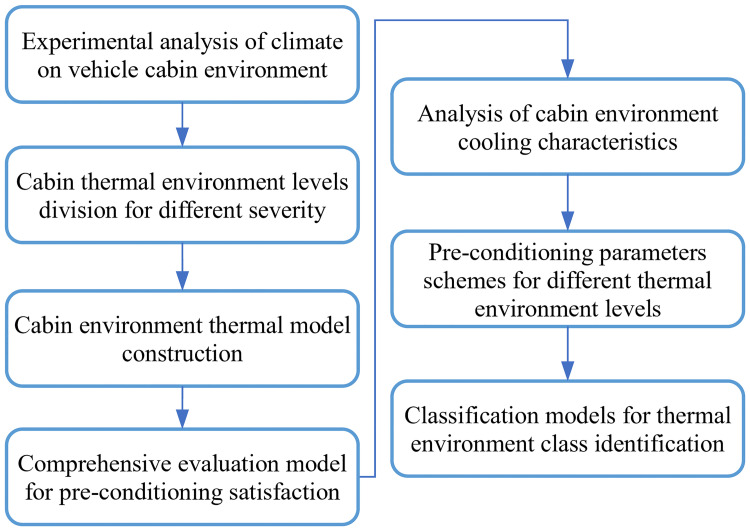
Research method of automobile cabin pre-conditioning. The system levels and classifies thermal environment. The database develops solutions for each thermal environment level. Cabin pre-conditioning invokes solutions based on the identified thermal environment level.

All differences in geographic, spatial, and temporal dimensions, as well as time and seasons variations, lead to differences in the impact of weather on the cabin environment. It is laborious and inefficient to develop an optimal solution of pre-conditioning for each condition needing multiple iterative operations. Therefore, the study constructs a database of thermal environmental variables and classifies them with the research methods in Fig 2. In this way, it is only necessary to develop control strategies for each category level and call them from the database, without repeating the analysis. This data-driven method provides effective solutions and achieves promising results through data training and classification with a simple model structure and fast calculation.

The research ideas of cabin pre-conditioning parameters decision have two main parts: candidate schemes formulation and data-driven decision model construction. On the one hand, typical climate scenarios from the experimental environment data set are selected to level multiple climate scenes. Parameter combinations of pre-conditioning are designed for corresponded levels with a proposed comprehensive evaluation index. On the other hand, the study suggests a classification algorithm model for identifying the climate level for the inputted environmental conditions and matching a suitable pre-conditioning parameters scheme.

**Fig 3:**
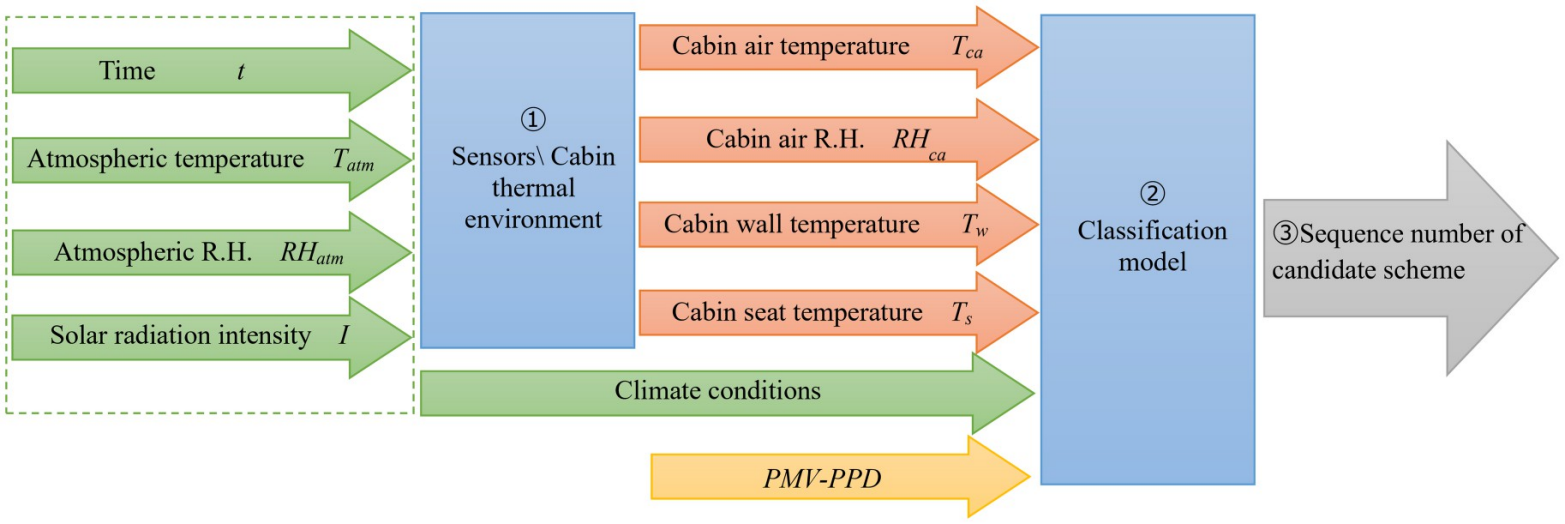
The data flow within the data-driven decision model. A classification model identifies and matches schemes based on the cabin temperature and humidity, seat and wall temperature and climate conditions.

The system obtains the thermal environment states in an automotive cabin coupling experimental climate data and machine learning algorithms. The data-driven decision model matches satisfactory cooling solutions according to hot severity levels of the thermal environments with variables relationship in Fig 3.

### Comprehensive evaluation index proposed for human multi-satisfaction

The effect factors on the passenger satisfaction of cabin cooling is varied, especially the high-temperature inner walls and poor air quality inside. A suitable pre-conditioning scheme should meet passenger’s multiple satisfaction needs. However, the PMV model focuses on the thermal state of the air. Therefore, a comprehensive evaluation index (*CEI*), new subjective evaluation method, is proposed to characterize the passengers’ satisfaction on considering multi-satisfaction objectives. The proposed *CEI* combines the thermal comfort, surface-cooling state, air quality, and energy consumption as represented by Eq (1),

$$\begin{array}{c} CEI=k_{TC}C_{TC}+k_{s}C_{s}+k_{a}C_{a}+k_{p}C_{p}, \end{array}$$ (1)

where *C*_*TC*_ is the thermal comfort scores; *C*_*s*_ is the surface acceptance scores; *C*_*a*_ is the air quality scores; *C*_*p*_ is the energy consumption scores; *k*_*TC*_, *k*_*s*_, *k*_*a*_, *k*_*p*_ are the weights coefficient of different items respectively. In this study, *k*_*TC*_ = 0.35, *k*_*s*_ = 0.2, *k*_*a*_ = 0.15, *k*_*p*_ = 0.3 that thermal comfort improving is prioritize and energy-saving is second. Compared with air quality, touching the seat and steering wheel in hot is more sensitive to human skin.

(1) Thermal comfort

The thermal comfort scores depend on the passenger’s thermal sensation and thermal comfort. These can be described by PMV-PPD indexes in EN ISO 14505 criterion, which are well-known variables. The global thermal sensation of people is divided into 7 levels, from -3 (cold) to 0 (neutral) to 3 (hot). These parameters are calculated as shown by Eqs (2) and (3) [26],

$$\begin{array}{cc} \text{PMV}= & (0.303e^{-0.036M}+0.028)[\left(M-W\right)-0.42\left[\left(M-W\right)-58.15\right]\\ & -3.05\times [5.733-0.007\left(M-W\right)-P_{ca}]-1.7\times 10^{-5}M\left(5867-Pa\right)\\ & -0.0014M(34-T_{ca})-3.96\times 10^{-8}f_{cl}[{\left(T_{cl}+273\right)}^{4}-{\left(T_{r}+273\right)}^{4}]\\ & -f_{cl}h_{c}(T_{cl}-T_{ca})] \end{array}$$ (2)

$$\begin{array}{c} \text{PPD}=100-95e^{\left(-0.03353PMV^{4}-0.2179PMV^{2}\right)} \end{array}$$ (3)

where *M* is the metabolism (W/m^2^); *W* is the human body work (W/m^2^), considering that the passenger walks into the car with 116 W/m^2^; *P*_*ca*_ is the cabin air pressure (Pa); *P*_*a*_ is the partial pressure of water vapor around the body, (kPa); *f*_*cl*_ is the clothing area factor; *T*_*cl*_ is the clothes temperature (°C); *T*_*r*_ is the radiation temperature (°C); *h*_*c*_ is the convection coefficient (Wm^−2^K^−1^).

Relevant researches concluded that the human is comfortable and satisfied with the thermal environment in which PPD < 10 [27]. With the increase of PPD, thermal conditions can lead to increased discomfort for occupants. Therefore, the inverse ratio function is used to determine the relationship between PPD and *C*_*TC*_, and the value of *C*_*TC*_ is normalized from 0 to 1. The thermal comfort scores *C*_*TC*_ is proposed as follows [27],

$$\begin{array}{c} C_{TC}=\{\begin{array}{ccc} -\frac{1}{30}\left(\text{PPD}-10\right)+1 & , & \text{PPD}>10\\ 1 & , & 0<\text{PPD}D<10 \end{array} \end{array}$$ (4)

(2) Surface-cooling state

The surface temperature of the seat in narrow cabins is a local thermal condition that profoundly affects passenger. The hot surface would cause discomfort to the tactile sensation of passengers, and even skin-damaging during prolonged exposure. Medical clinical studies found that 42°C is the critical temperature to cause moderate temperature burns and over 70°C will cause skin high-temperature burns [28–30]. Therefore, during the cabin pre-conditioning, the temperature of the human main contact surface should decrease lower than 42°C to avoid skin moderate temperature burns. As the seating surface is the main contact area for passengers which absorbs a lot of heat, the satisfaction on seat state is necessary to be assessed. The model set 40°C as the critical temperature of satisfactory surface cooling down considering medical research [31] and the satisfaction increases as the surface cooling, so the *C*_*s*_ is proposed as,

$$\begin{array}{c} C_{s}=\{\begin{array}{c} 0\;,40^{{}^{\circ}}C\leq \text{max}\{T_{s1},T_{s2}\}\\ -\frac{T_{s,\text{max}}}{20}+2,20^{{}^{\circ}}C\leq \text{max}\{T_{s1},T_{s2}\}\leq 40^{{}^{\circ}}C\\ 1\;,\text{max}\{T_{s1},T_{s2}\}\leq 20^{{}^{\circ}}C \end{array} \end{array}$$ (5)

(3) Air quality

The releasing of harmful substances from car interiors is accelerated by high temperature conditions, harming the occupants’ physical health [32]. The external circulation is the main way of cabin ventilation during cooling, impacting the air quality significantly [33]. The air circulation rate *β* is a parameter to control the mixing ratio of ambient air and cabin circulating air. A low circulation rate *β* can obtain a high percentage of outside fresh air. Therefore, the air quality scores *C*_*a*_ in *CEI* is proposed as,

$$\begin{array}{c} C_{a}=1-\beta \end{array}$$ (6)

(4) Energy consumption

Actuality, the energy consumption of HVAC is calculated by the performance compression curve of the compressor and the change in enthalpy actuality. To simplify the calculation, the study approximates that energy consumption is inversely proportional to the HVAC outlet airflow temperature *T*_*ac*_ [18]. High energy consumption would reduce users’ satisfaction. With normalization, the energy consumption satisfaction item is proposed as,

$$\begin{array}{c} C_{p}=14/(2.4T_{R}) \end{array}$$ (7)

where compressor power *P*_*c*_ is 14 kW; normalized coefficient *n*_*p*_ is 2.4.

### Model for cabin thermal environment

Generally, the airflow and other local surfaces within the cabin have different thermal responses to various levels of environmental loads. Therefore, it is a premise of cabin pre-conditioning strategy formulation to analyze the impact of environmental factors and supply air characteristics on the cabin thermal characteristics. A model for cabin thermal environment is established to determine the thermal environment characteristic and cabin pre-conditioning effects of the considered schemes. Combining with the proposed *CEI*, suitable solutions for various climate levels are explored.

(1) Thermal transfer between vehicle cabin and its surroundings

**Fig 4:**
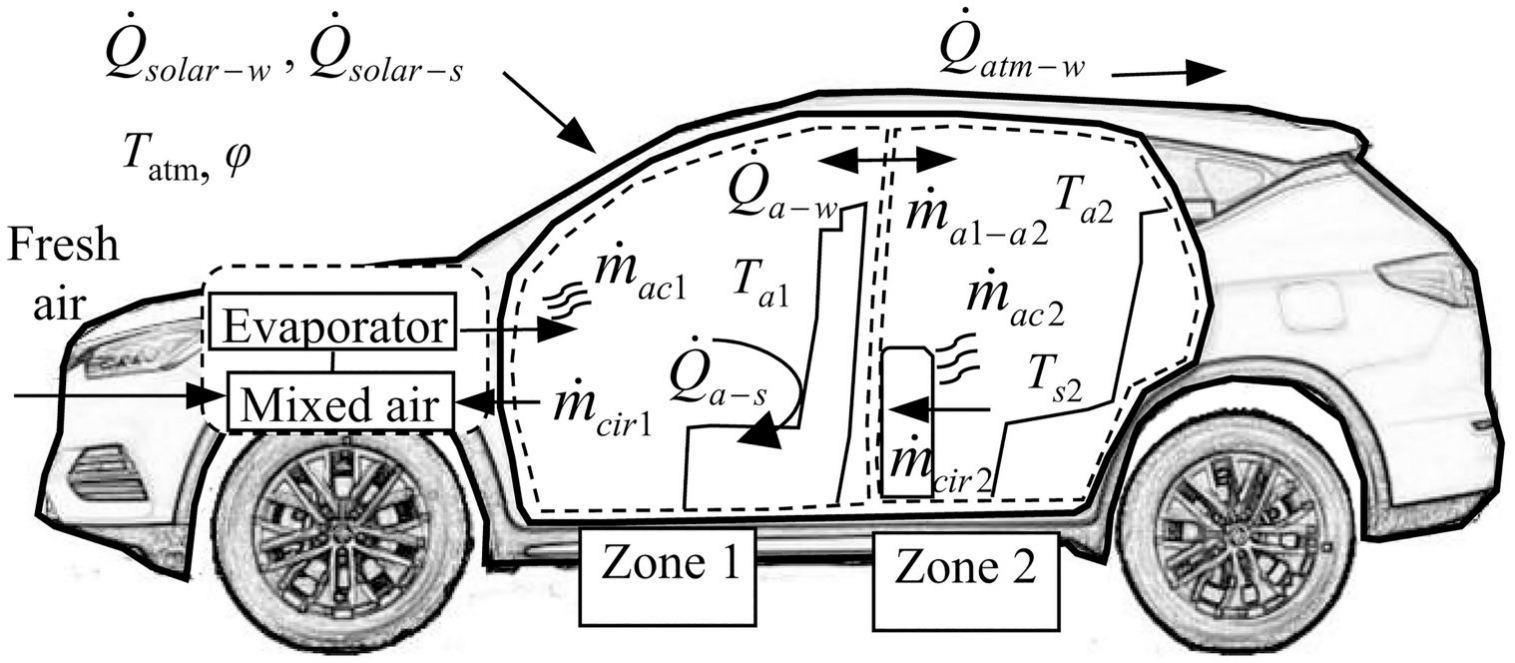
The physical relationship of cabin thermal transfer. Solar radiation (Δ*Q*_solar −*w*_, Δ*Q*_solar −*s*_) is an important factor to the cabin temperature, the convection of external airflow (Δ*Q*_*atm*−*s*_) as well. As the uneven airflow from HVAC, the cabin is divided into two thermal zones: the front row’s region (zone 1) and the rear row’s region (zone 2). During cooling, thermal zones are affected by energy and mass exchanges: 1) the convection (Δ*Q*_*a*−*w*_, Δ*Q*_*a*−*s*_) and cooling mass flow (Δ*m*_*ac*_) from air-conditioning; 2) the solar radiation load (Δ*Q*_solar −*w*_, Δ*Q*_solar −*s*_) received; 3) air circulation between zones (Δ*m*_*a*1−*a*2_) and in HVAC (Δ*m*_*cir*_); 4) thermal exchanges with outside (Δ*Q*_*atm*−*s*_).

The external environment affects the cabin’s thermal environment through convection and solar radiation. And the air supplied from the HVAC system makes the dynamic non-uniform change of the cabin thermal environment more intense. To study the cooling effects of different air conditions, the model of the thermal transfer in the cabin is built in Fig 4. According to the heat transfer relationship of the cabin, a thermal model is constructed to analyze the cabin thermal environment.

(2) Energy balance in the cabin

The cabin thermal model is performed with Lumped Capacitance Method. The temperature distribution in each thermal zone is assumed to be uniform. Dynamic equations are constructed to calculate the air temperature and humidity in the cabin, including the mass balance equation and energy balance equation.

The mathematical model is derived from the energy conservation and mass balance in thermal zone 1 and 2. The heat exchange of air, wall, and seat are both considered. The equation can be given as follow, while the specific heat capacity *c*_*i*_ of the air is set as a constant. The distribution ratio *δ* is the proportion of air from the vents in the dashboard (zone 1) that directly enters the rear area (zone 2).

$$\begin{array}{c} \frac{dT_{ca1}}{\;dt}=\frac{\Delta Q_{ca1}+\delta \Delta m_{ac1}c_{ca}(T_{ac1}-T_{ca1})-\Delta m_{ca1-ca2}c_{ca}(T_{ca1}-T_{ca2})}{c_{ca}m_{ca1}} \end{array}$$ (8)

$$\begin{array}{cc} \frac{dT_{ca2}}{\;dt}= & \frac{\Delta Q_{ca2}+\left(1-\delta \right)\Delta m_{ac1}c_{ca}(T_{ac1}-T_{ca2})+\Delta m_{ac2}c_{ca}(T_{ac2}-T_{ca2})}{c_{ca}m_{ca2}}\\ & +\frac{\Delta m_{ca1-ca2}c_{ca}(T_{ca1}-T_{ca2})}{c_{ca}m_{ca2}} \end{array}$$ (9)

$$\begin{array}{c} \frac{dT_{s1}}{\;dt}=\frac{\Delta Q_{solar-s1}+\Delta Q_{ca1-s1}-\Delta Q_{s1-w1}-\Delta Q_{s1-w2}}{c_{s}m_{s1}} \end{array}$$ (10)

$$\begin{array}{cc} \frac{dT_{w1}}{\;dt}= & \frac{\Delta Q_{\text{solar}\;-w1}+\Delta Q_{ca1-w1}+\Delta Q_{atm-w1}+\Delta Q_{w2-w1}}{c_{w}m_{w1}}\\ & -\frac{\Delta Q_{w1-s1}-\Delta Q_{w1-s2}-\Delta Q_{w1-sky}}{c_{w}m_{w1}} \end{array}$$ (11)

*Q*_*w*1−*w*2_ is thermal conduction of the body wall between zone 1 and zone 2. The thermal convective load *Q*_*ca*_ affects the air temperature of the cabin. The seats and walls receive the gain *Q*_solar −*w*_, *Q*_solar −*s*_ of solar radiation while radiating their own heat *Q*_*w*−*s*_, *Q*_*s*−*w*_ to the surroundings and cooling with convection *Q*_*ca*−*w*_, *Q*_*ca*−*s*_. The calculation method of the above thermal load can be obtained in the literature [17, 21].

(3) Moisture mass balance in the cabin

The moisture mass in the cabin is affected by the water vapor content from HVAC. Regarding the cabin as the control volume, the moisture balance can be expressed as,

$$\begin{array}{c} \frac{dw_{ca1}}{\;dt}=\frac{\delta \Delta m_{ac1}(w_{ac1}-w_{ca1})-\Delta m_{ca1-ca2}(w_{ca1}-w_{ca2})}{m_{ca1}} \end{array}$$ (12)

$$\begin{array}{cc} \frac{dw_{a2}}{\;dt}= & \frac{\left(1-\delta \right)\Delta m_{ac1}(w_{ac1}-w_{ca2})+\Delta m_{ac2}(w_{ac2}-w_{ca2})}{m_{ca2}}\\ & +\frac{\Delta m_{ca1-ca2}(w_{ca1}-w_{ca2})}{m_{ca2}} \end{array}$$ (13)

The fresh air and circulating air are mixed to enter the evaporator with an enthalpy *i*_*mix*_. The energy conservation and mass balance occur in the evaporator, and supplied air enthalpy *i*_*ac*_ can be expressed [17],

$$\begin{array}{c} (\Delta m_{ac1}+\Delta m_{ac2})w_{m\dot{x}}=(\Delta m_{ac1}+\Delta m_{ac2})w_{m\dot{x}}+\Delta m_{wa} \end{array}$$ (14)

$$\begin{array}{c} i_{ac}=i_{mix}-\frac{\Delta Q_{evap}}{\left(\Delta m_{ac1}+\Delta m_{ac2}\right)}-\frac{m_{wa}i_{wa}}{\left(\Delta m_{ac1}+\Delta m_{ac2}\right)} \end{array}$$ (15)

where *m*_*wa*_ is the mass of water condensed on the cooling surface of the evaporator.

### Classification model for environment levels identification

Identifying the current thermal environment level of the cabin is a prerequisite for targeted and satisfactory pre-conditioning work. The research classifies the real-time weather conditions and the cabin temperature with classification algorithms to clarify which level of the cabin environment is in. The classification algorithm determines a reasonable parameter scheme based on the cabin climate level. The algorithms obtain objective classification rules through the analysis of the training set of known categories, mapping each attribute set *x* to a pre-defined class label *y*, to predict the category of the new data. The study surveys Support Vector Machines (SVM) [34], K-nearest neighbor (KNN) [35], and Decision Tree algorithms [34] for thermal environment levels identification when cabin pre-conditioning on. The selected methods are well established and their basic principles can be obtained from the relevant literature. By comparing the effectiveness of the methods, the method with the highest accuracy in identifying the thermal environment class is selected.

## Thermal environment characteristic experiments for parked vehicles

The pre-conditioning strategy uses a hierarchical approach to rate the thermal states in the cabin, so it is required to clarify how the thermal states are caused by climate conditions. The climate conditions, such as solar radiation, ambient air temperature and humidity, vary when vehicles are parked in open-space, shade and underground. Cars in open parking have the most comprehensive range of environmental parameters experienced by vehicles. Therefore, the study experimentally investigated the cabin thermal environment characteristics formed by climate conditions for an open parked vehicle. The experiment provides support for rating the thermal environment levels and model verification.

### Experimental design and data collection

The tested vehicle is a sport utility vehicle (SUV) with five doors, black. The front end of the vehicle faces the south without any shelter to ensure the most severe thermal environment inside the car. The natural climate exposure experiment was carried out in Jeddah, Saudi Arabia (21°3’ N, 39°1’ E), in a tropical desert climate. The duration of the experiment was for a whole year, from March 2016 to February 2017.

**Fig 5:**
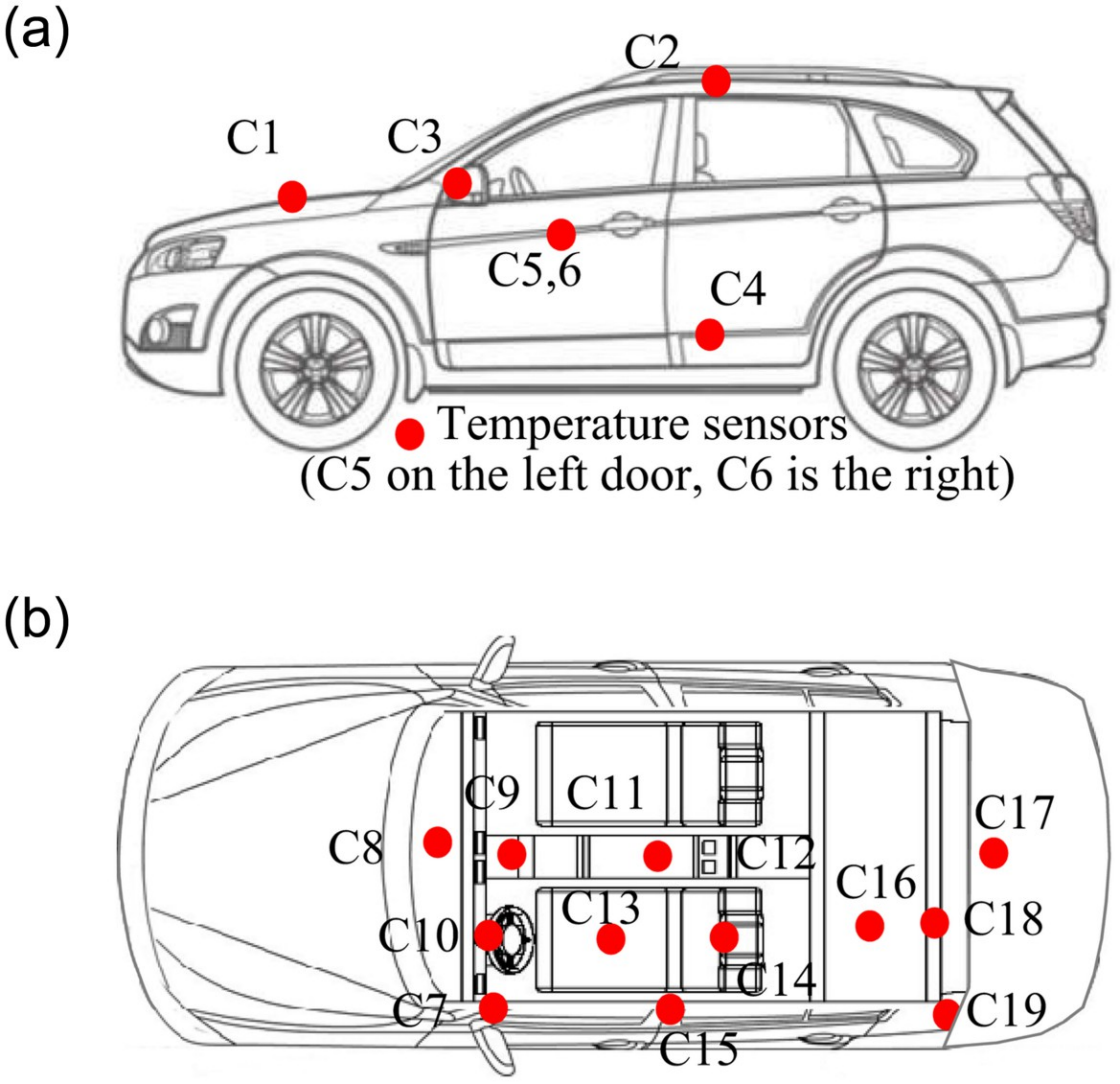
Arrangement of thermocouples in the cabin. The temperature measurement points on the car body were set to analyze the thermal load of the vehicle. (a): Measurement points on the car body. (b): Measurement points in the cabin.

**Fig 6:**
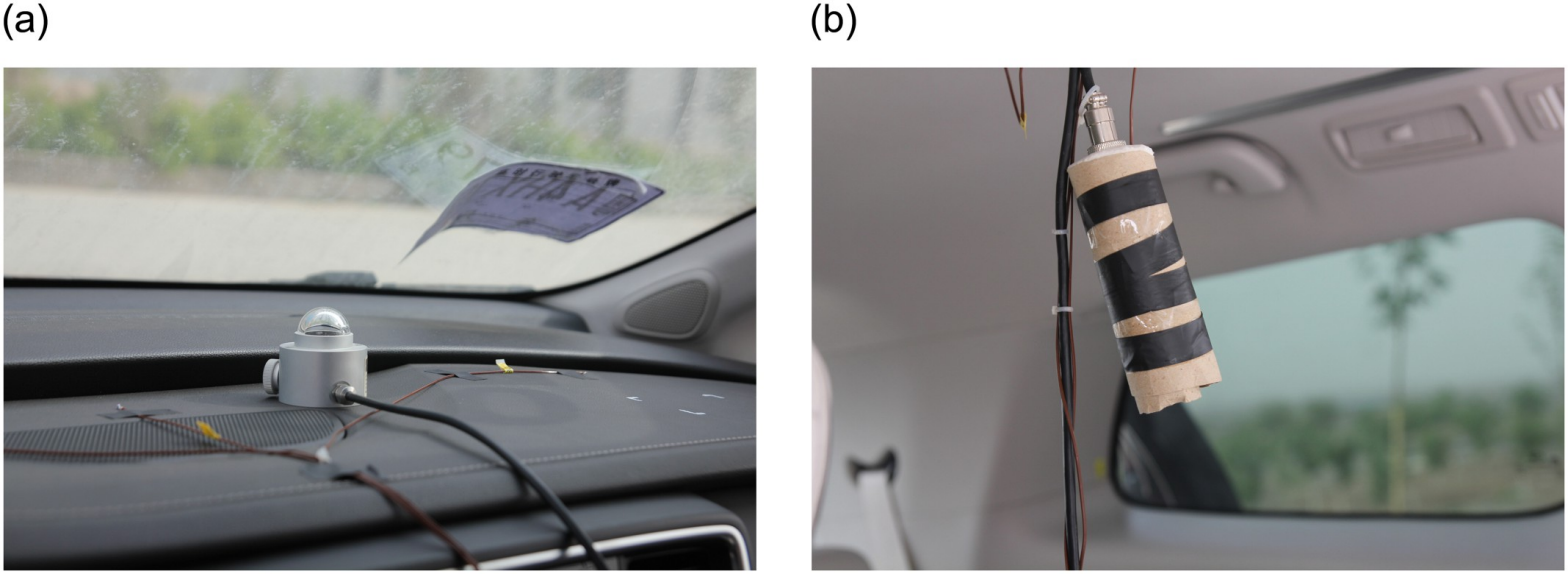
Experimental scenario and related layout. A solar radiation meter was set to obtain the amount of solar radiation entering the cabin through the windows. Air temperature and humidity were measured by a sensor suspended from the roof. (a): Dashboard’s thermocouples and radiometer. (b): Air temperature and humidity sensor.

The experiment examined air temperature, humidity, precipitation, total solar radiation of ambient environment and cabin environment, the thermal characteristic of vehicle structural as well. Ambient environmental parameters were collected via a meteorological test platform. The cabin temperature data of wall surfaces were logged using T-type thermocouples (± 0.1°C) with the Agilent 34970A data collector. The temperature measurement points on the car body were set as shown in Fig 5. The case study measured the variables of solar radiation, atmosphere temperature, and relative humidity. The sensors were installed as shown in Fig 6.

### Experimental results

The measured parameters were recorded every 5 minutes, and a total of 104,955 sets of valid experimental data were obtained from the experiment. Experiments were conducted to obtain the characteristics of the thermal load on the cabin during parking in a tropical desert climate. It has a clear picture of the maximum and minimum temperature states that exist in the vehicle, helping to define and differentiate levels of thermal environments.

**Fig 7:**
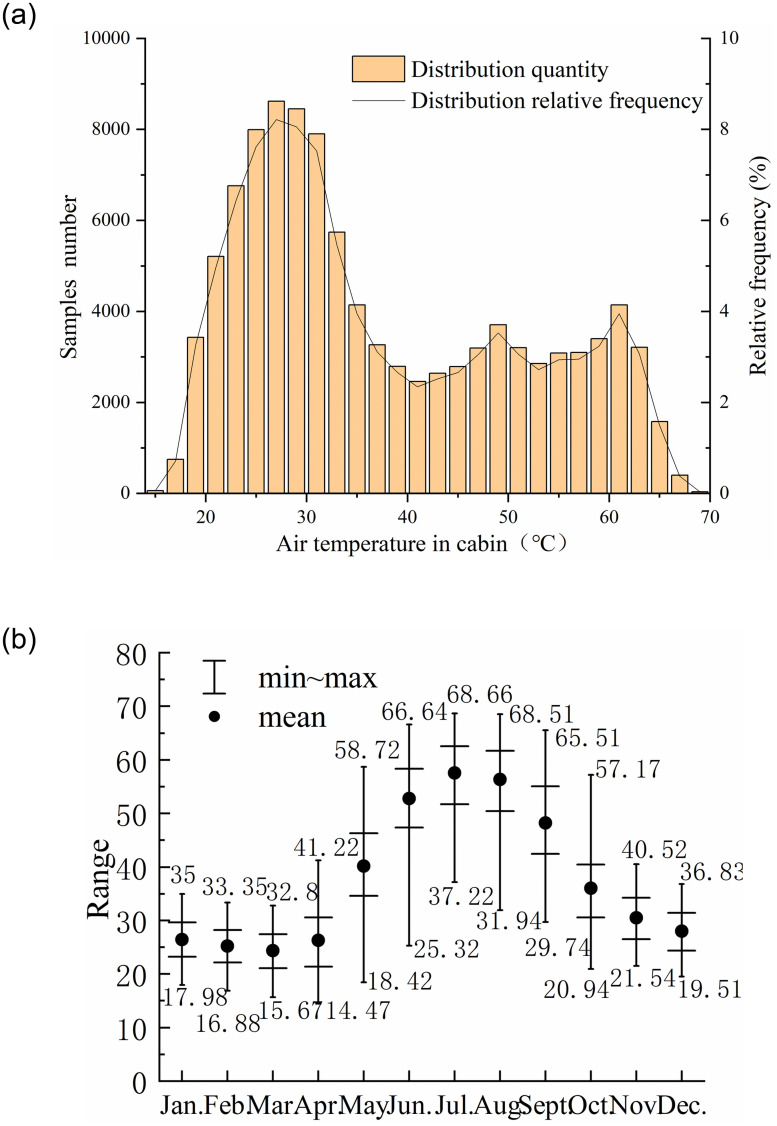
Cabin air temperature *T*_*ca*_ obtained by experiment. The frequency in (a) is described as $f\left(T_{ca}\right)=8.331e^{-{\left(\frac{T_{ca}-27.33}{7.989}\right)}^{2}}+3.447e^{-{\left(\frac{T_{ca}-52.75}{14.7}\right)}^{2}}$; while average cabin temperature *f*(*t*_*m*_) = 48.08 sin(0.172*t*_*m*_ + 0.257)+ 11.06 sin(0.86*t*_*m*_ + 1.65). *t*_*m*_ is the month. (a): Frequency of occurrence of cabin air temperature. (b): Change in cabin air temperature by month.

Under the tropical desert climate, the vehicle cabin was at a high temperature most of the time. The statistics of the air temperature in the cabin are shown in Fig 7. From Fig 7(a), the range from 27°C to 28°C has the most samples, accounting for 8.3%. The proportion of air temperature over 26°C accounts for 73%, which means that the thermal environment of the cabin is unsatisfactory most time. From June to September, cabin air temperature both exceeded 25°C, and even in winter exceeds 30°C under sun exposure in Fig 7(b), confirming the necessity for cabin pre-conditioning.

**Fig 8:**
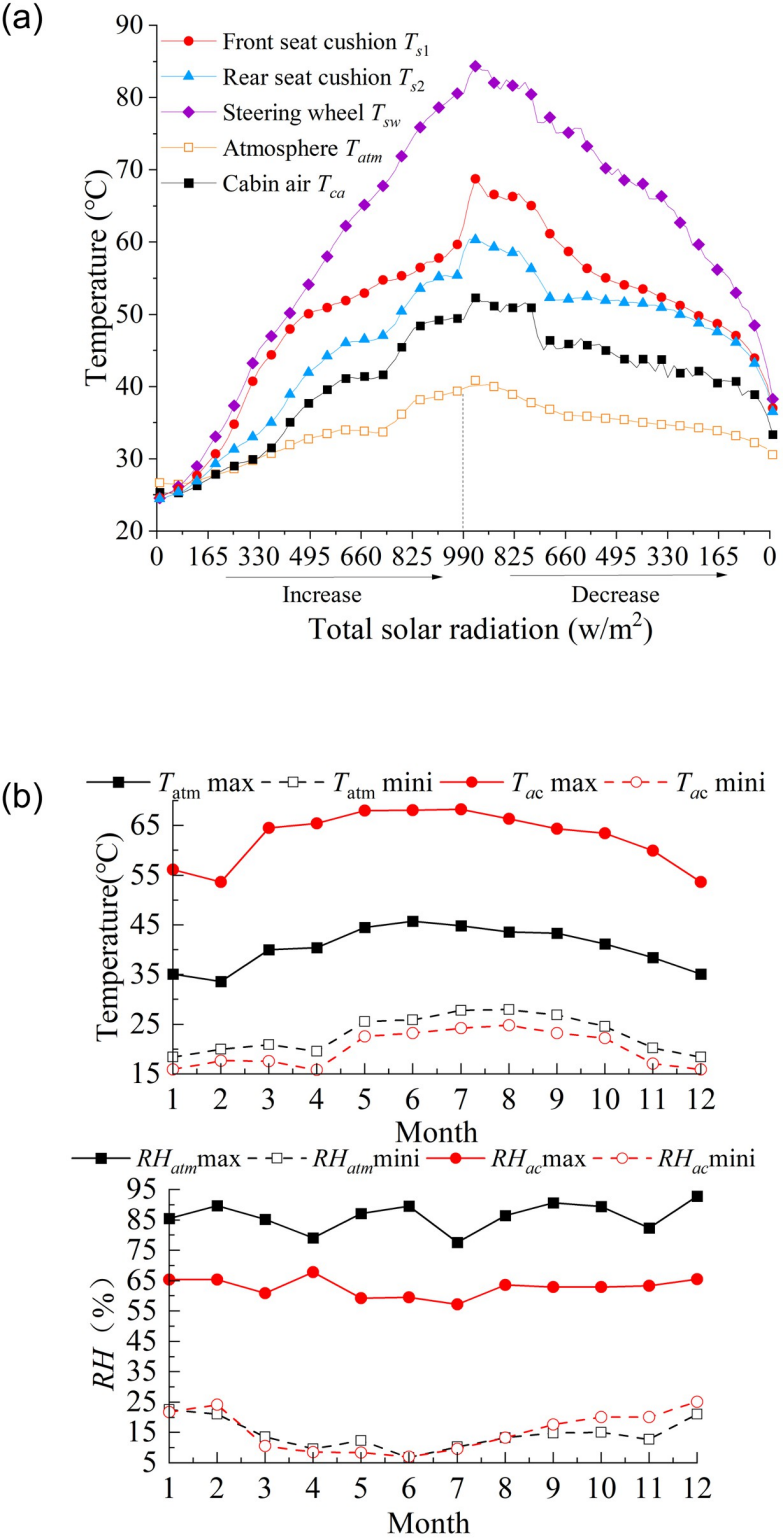
Thermal load of main components during parking. During the radiation increasing, the relationship between the solar radiation and the components temperature follows as: atmosphere, *T*_*atm*_ = 0.006086*I*^1.117^ + 25.95; cabin air, *T*_*ca*_ = 0.006086*I*^1.117^ + 25.95; the steering wheel, *T*_*Sw*_ = 0.1161*I*^0.9124^ + 20.69; front seat, *T*_*s*1_ = 2.475*I*^4^ + 0.3333*I*^3^−9.094*I*^2^ + 10.64*I* + 50.25; rear seat, *T*_*s*2_ = −0.1648*I*^2^ + 10.35*I* + 40.8; During the radiation decreasing, the correlation follows as: atmosphere $T_{atm}=11.41e^{-{\left(\frac{I-1.879}{0.9813}\right)}^{2}}+35.09e^{-{\left(\frac{I+0.1619}{4.424}\right)}^{2}}$; steering wheel *T*_*Sw*_ = 1.739*I*^0.4881^ + 33.89; front seat *T*_*s*1_ = −1.886*I*^4^ + 0.8018*I*^3^ + 4.752*I*^2^ + 6.391*I* + 53.98; rear seat *T*_*s*2_ = −0.8943*I*^4^ + 1.891*I*^3^ + 1.865*I*^2^ + 1.704*I* + 51.24. In (b), the atmospheric temperature is described as $T_{atm\;\text{max}}=44.92e^{-{\left(\frac{t_{m}-6.836}{10.53}\right)}^{2}}$ and $T_{atm\;\text{min}}=26.84e^{-{\left(\frac{t_{m}-7.049}{8.572}\right)}^{2}}$; cabin air temperature are $T_{ac\;\text{max}}=68.4e^{-{\left(\frac{t_{m}-6.547}{11.44}\right)}^{2}}$ and *T*_*ac*min_ = $23.58e^{-{\left(\frac{t_{m}-7.099}{8.336}\right)}^{2}}$; atmospheric *RH* are described as *RH*_atm max_ = $0.1285t_{m}^{2}-1.365t_{m}+88.21$ and $RH_{atm}\;\text{min}=0.3778t_{m}^{2}-5.078t_{m}+26.98$; cabin *RH* are described as $RH_{ac\;\text{max}}=0.1679t_{m}^{2}-2.243t_{m}+68.3$ and $RH_{ac\;\text{min}}=0.5135t_{m}^{2}-$ 6.219*t*_*m*_ + 28.13. (a): The influence of changes in solar radiation. (b): Maximum and minimum of air temperature and relative humidity.

For a vehicle parked in the open air, its thermal environment can be divided into three phases according to the changes in solar radiation: increasing radiation, decreasing radiation and none radiation. Fig 8(a) presents thermal characteristics of the cabin environment as solar radiation increases to decrease. It is worth noting that the highest cabin air temperature *T*_*ca*_ occurs at the beginning of the solar radiation waning, while the lowest is at the appearance of solar radiation, due to the thermal storage effect of the material. During decreasing radiation, the greenhouse effect maintains the occupant compartment at a higher temperature than the same level of solar radiation during radiation increase. And during the none radiation phase, the occupant compartment temperature continues to decrease to a minimum, close to the ambient temperature with thermal equilibrium, shown in Fig 8(b). The *RH* max occurs at the lowest temperature and the *RH* mini occurs at the highest temperature.

**Fig 9:**
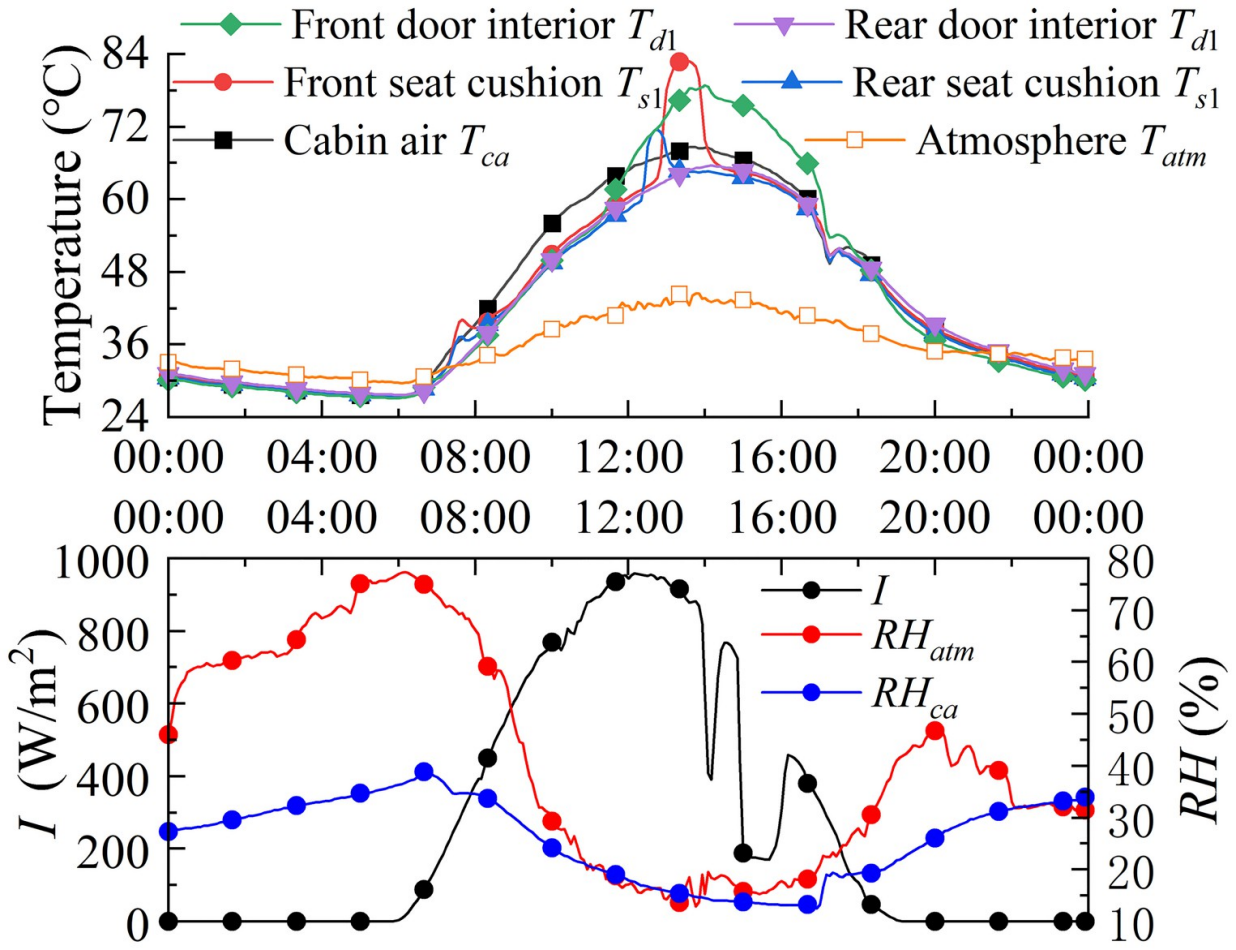
Temperature and humidity distribution on July 15. The car suffered from the worst heat load at noon, with a maximum *T*_*ca*_ of 68.66°C.

The highest temperature measured in the crew cabin during the experiment was on 15 July, as shown in Fig 9. The experimental results in Fig 9 show that the air temperature in the cabin increases with the atmosphere due to the solar radiation. Due to the thermal insulation effect of the car interior, the cabin air temperature remains at a relatively high level in the early part of the evening. The state of time is therefore also an influencing factor for the cabin environment to be considered.

## Results and discussion

### Environmental levels division for pre-conditioning scenario

**Fig 10:**
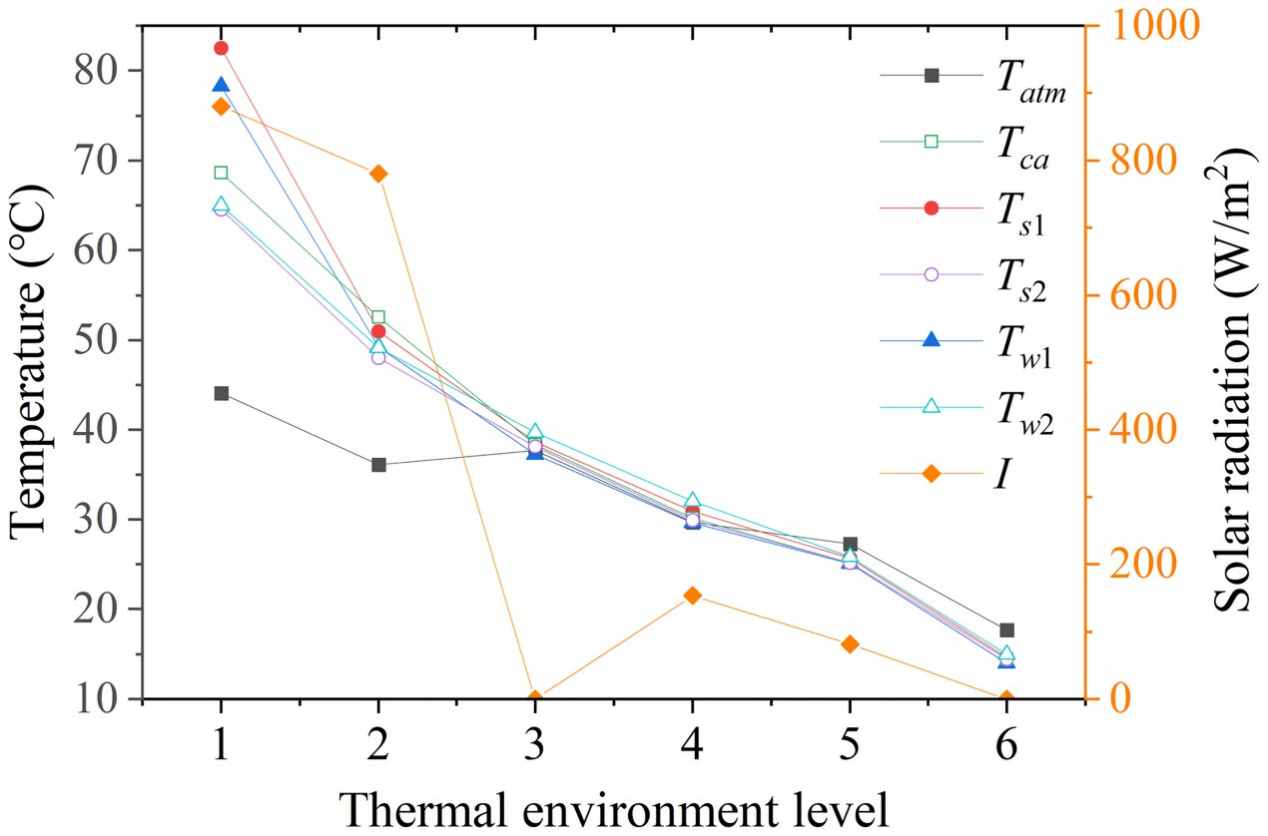
Characteristics of scenarios for different thermal environment levels. *T*_*ca*_ and *RH*_*ca*_ are respectively the air temperature and relative humidity of the cabin, measured at the front and rear rows. *T*_*s*1_ and *T*_*s*2_ are the temperatures of the front and rear seat cushion, while *T*_*w*_ is derived from the average wall temperature in the thermal zone, including car door interior trim panel, pillars, the car roof, and floor.

Natural environmental conditions are variable and uncertain. This system levels multiple climate scenes corresponding to variables in the cabin air temperature and provides them with several tailored pre-conditioning solutions. Based on experimental results, the thermal environment levels in the vehicle cabin are graded by the magnitude of the cabin air temperature *T*_*ca*_ to satisfy most of the real scenarios. The experimental data of *T*_*ca*_ are ranked in descending order and classified into 6 levels of thermal environment varied from hot, warm to neutral levels according to the average intervals. Representative specify thermal environment scenarios are selected to character environment levels in Table 1 and Fig 10 considering the differences in solar radiation intensity *I* and ambient temperature *T*_*atm*_ over time.

**Table 1:** The typical scenarios for different thermal environment levels.

Level	Date	*T*_*atm*_ (°C)	*RH*_*atm*_ (%)	*I* (W / m^2^)	*T*_*ca*_ (°C)	*RH*_*ca*_ (%)	*T*_*s*1_ (°C)	*T*_*s*2_ (°C)	*T*_*w*1_ (°C)	*T*_*w*2_ (°C)	PMV	PPD
**1**	2016/07/15 13:40	44.12	15.04	880.00	68.66	14.95	82.50	64.54	78.31	65.00	3.00	100.00
**2**	2016/08/03 10:20	36.13	51.64	780.90	52.57	33.2	50.98	48.01	49.21	49.15	3.00	100.00
**3**	2016/06/24 20:25	37.69	12.45	0.00	38.39	11.38	38.70	38.12	37.26	39.70	2.07	79.95
**4**	20160/9/28 07:25	29.72	82.90	153.70	30.13	61.50	30.88	29.9	29.63	32.04	1.67	60.05
**5**	2016/10/26 07:15	27.26	73.95	81.10	25.20	54.36	25.66	25.18	25.11	25.83	1.10	30.47
**6**	2017/02/18 07:35	17.67	43.97	0.04	14.47	49.96	14.60	14.45	14.01	14.98	-0.15	5.48

Six levels indicate various pre-conditioning requirements of the cabin environment. The first two scenarios mainly occur in the afternoon with high solar radiation, while the latter two are mainly during the morning. The study sets up a simulation analysis to explore the suitable pre-conditioning schemes corresponding to these 6 thermal environmental levels.

### Pre-conditioning schemes for various thermal environment levels

#### Requirements and objectives for cabin pre-conditioning

The developed cabin pre-conditioning schemes should achieve a satisfactory thermal environment within the specified time (10 minutes) using as low energy consumption as possible. External fresh air also should be considered to minimize the toxic gas content accumulated. This allows passengers to be as comfortable as possible in the cabin when they enter. Generally, it takes about 10 minutes for citizens to travel from their workplaces to their parking places [36]. Therefore, the pre-conditioning schemes should meet the two objectives. Firstly, the cabin pre-conditioning should be completed within 10 minutes; secondly, the *CEI* of the thermal environment after cabin pre-conditioning should be high as possible that satisfy passengers.

#### Boundary conditions

The dynamic thermal responses during cooling are simulated with the established model of cabin thermal environment under various conditions and control strategies. In simulations, the settings of vehicle dimensions were taken from manufacturer data, 3100mm × 1700mm × 1300mm cabin sizes, 180ml HVAC compressor. The major material parameters including the thermodynamic and optical properties were consistent with previous related research [5, 37, 38]. The environmental parameters in Table 1 were used as boundary conditions for the different environmental levels. The external environmental factors were simplified as a constant value during the pre-conditioning.

The cooling performance of the HVAC parameter combinations was analyzed sequentially for each thermal environment level. The simulations conditions respectively selected a value from the candidate ranges of *T*_*R*_, *v*_*ac*_, and *β* shown in Table 2 to form a parameter combination [*T*_*R*_, *v*_*ac*_, *β*] for cabin pre-conditioning. The refrigerant temperature *T*_*R*_, velocity of supplied air *v*_*ac*_, and air circulation rate *β* are used as the control variables of HVAC to control the *T*_*ca*_ and Δ*m*_*ca*_. The *T*_*R*_ depends on the performance compression curve of the HVAC compressor, so *T*_*R*_ needs an electronic control of the compressor to be managed.

**Table 2:** The typical scenarios for different thermal environment levels.

Level	*T*_*R*_ (°C)	*v*_*ac*_ (m/s)	*β* %
**1**	6, 8, 10	7.5, 4.5, 1.5	100, 75, 50, 25, 0
**2**	6, 8, 10, 12	7.5, 4.5, 1.5	100, 75, 50, 25, 0
**3**	6, 8, 10, 14	7.5, 4.5, 1.5	100, 75, 50, 25, 0
**4**	8, 10, 12, 14	7.5, 4.5, 1.5	100, 75, 50, 25, 0
**5**	10, 12, 14	7.5, 4.5, 1.5	100, 75, 50, 25, 0
**6**	12 14, ∖	7.5, 4.5, 1.5	100, 75, 50, 25, 0

(Note: The ‘∖’ means that HVAC is off for cooling, and only the blower blows.).

The velocity of supplied air selected in Table 2 corresponds to the blower power *P*_*b*_ being 100 W, 60 W, and 20 W. It is worth noting that when setting *β*=100%, *β*=0% is used first for 60 seconds, after which *β*=100% takes effect. This setup is to improve the air quality and reduce the toxic gas content in the cabin as possible.

#### Parameter schemes for different thermal environment levels

To meet multi satisfaction objectives, the *CEI* proposed is applied to evaluate the satisfaction on various cabin pre-conditioning schemes. Through multiple simulations and comparisons with *CEI*, the research has formulated 6 pre-conditioning schemes for cabin thermal environment, as shown in Table 3. For Level 6, the airflow is supplied from the blower without cooling. Each combination scheme obtains the highest *CEI* index to match each scenario level. All the selected parameter schemes have excellent comprehensive performance and consider multi-satisfaction objectives that more realistically meet passengers’ needs for vehicle using.

**Table 3:** The typical scenarios for different thermal environment levels.

Level	[*T*_*R*_, *v*_*ca*_, *β*]	PPD in zone1	PPD in zone2	*CEI*
**1**	[6, 100, 100]	7.5, 4.5, 1.5	100, 75, 50, 25, 0	-0.099
**2**	[8, 100, 100]	7.5, 4.5, 1.5	100, 75, 50, 25, 0	0.190
**3**	[10, 100, 75]	7.5, 4.5, 1.5	100, 75, 50, 25, 0	0.372
**4**	[14, 60, 100]	7.5, 4.5, 1.5	100, 75, 50, 25, 0	0.385
**5**	[14, 20, 25]	7.5, 4.5, 1.5	100, 75, 50, 25, 0	0.398
**6**	[∖, 100, 0]	7.5, 4.5, 1.5	100, 75, 50, 25, 0	0.694

(Note: The ‘∖’ means that HVAC is off for cooling, and only the blower blows.).

**Fig 11:**
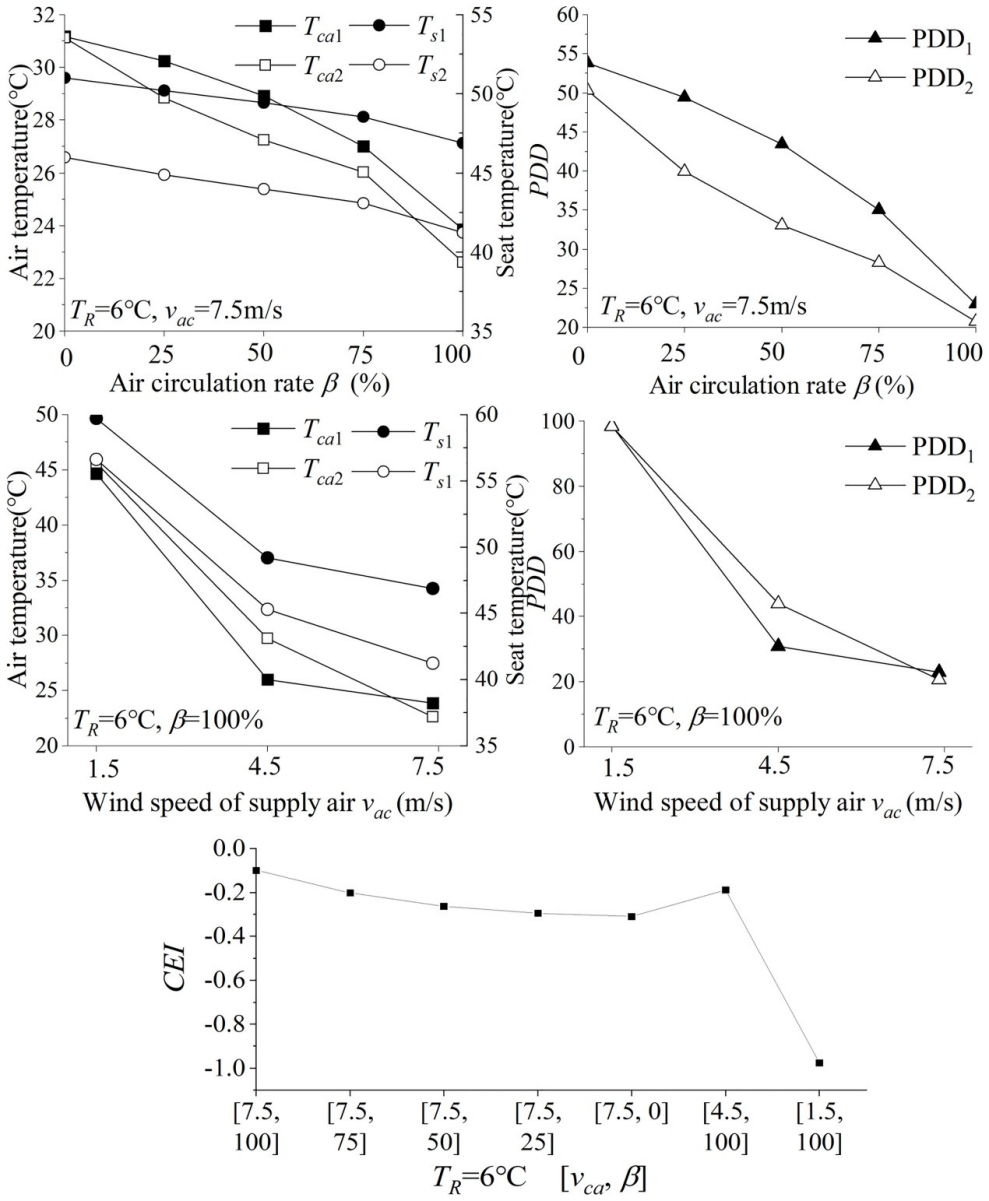
Influence of different parameter combinations on scenario 1. Compared with others, the parameter scheme [6, 7.5, 100] wins the highest score, with a *CEI* being -0.099.

During the scheme analysis of Level 1, the cooling effect of different parameter combinations is shown in Fig 11. As the *β* decreases, the cooling effect would decrease leading to a higher air temperature. The main reason is that external circulation improves air quality by introducing ambient air but the ambient heat is also introduced into the system. The mass flow rate of the supply air also affects the cooling effect. With a small supplied air volume, the thermal environment in the cabin is still hot with *T*_*ca*1_ being 44.6°C and *T*_*s*1_ being 59.7°C at 10 minutes. For Level 1 scenario, *T*_*ca*1_ is still over 24°C for any combinations. Also, *T*_*s*_ is still higher than 40°C, which will cause a slightly hot touch to the passengers. Limited by the performance of the HVAC system of a vehicle, a longer pre-conditioning time would be a more effective solution for more satisfactory cooling results for these extreme scenarios.

The parameters in Table 3 are conducted for satisfaction analysis. Level 1 has the most severe thermal environment experienced in the cabin. The *CEI* of all schemes are less than 0 for the Level 1 scenario, which means unsatisfied items still exist. Compared with other schemes, the parameter scheme [6, 7.5, 100] wins the highest score, with a CEI being -0.099 in Fig 11. Therefore, the scheme [*T*_*R*_=6°C, *v*_*ac*_=7.5m/s, *β*=100%] is the better choice to solve those thermal environments in Level 1.

**Fig 12:**
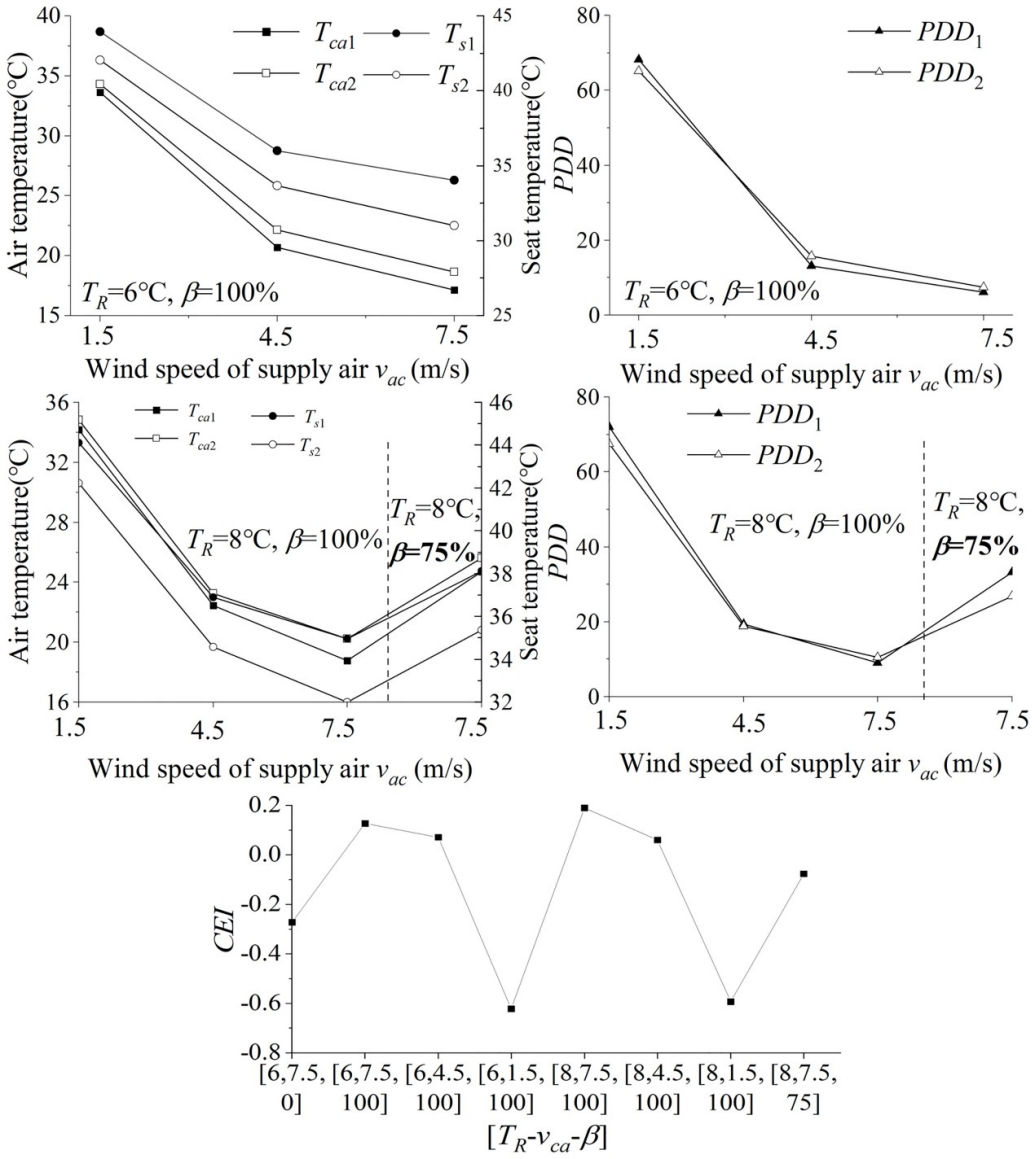
Influence of different parameter combinations in 7.5 m/s on scenario 2. The parameter combination [8, 7.5, 100] wins the higher score.

The cooling effect in 10 minutes of different combinations for Level 2 is presented in Fig 12. With constant *T*_*ac*_, the reduction of *β* would fresh the cabin air but increase *T*_*ca*_. With multiple groups of simulations, the parameter combinations [6, 7.5, 100] and [8, 7.5, 100] enable to make cabin environment satisfactory. As the air circulation rate increases, the PPD index of the thermal environment decreases. In combinations [6, 7.5, 100] and [8, 7.5, 100], the PPD of the front thermal zone is less than 10%, and the back row is also close to 10%.

According to the *CEI* model, the combination [8, 7.5, 100] wins the higher score, chosen as the pre-conditioning schemes to solve the thermal environment scenario in Level 2. Due to limited space, the simulation results of other scenarios are shown in Table A1 in S2 Appendix.

### Training and verification of decision model

To accurately and effectively classify thermal environment levels and pre-conditioning decisions, data-driven methods are applied to identify inputted environmental conditions. The classification algorithms: support vector machines (SVM), Decision Tree and Random Forest (RF), and weighted K-Nearest Neighbor (KNN) are compared in environment level identification. The predicted environmental level would be used to match the corresponding parameter scheme to achieve pre-conditioning.

**Fig 13:**
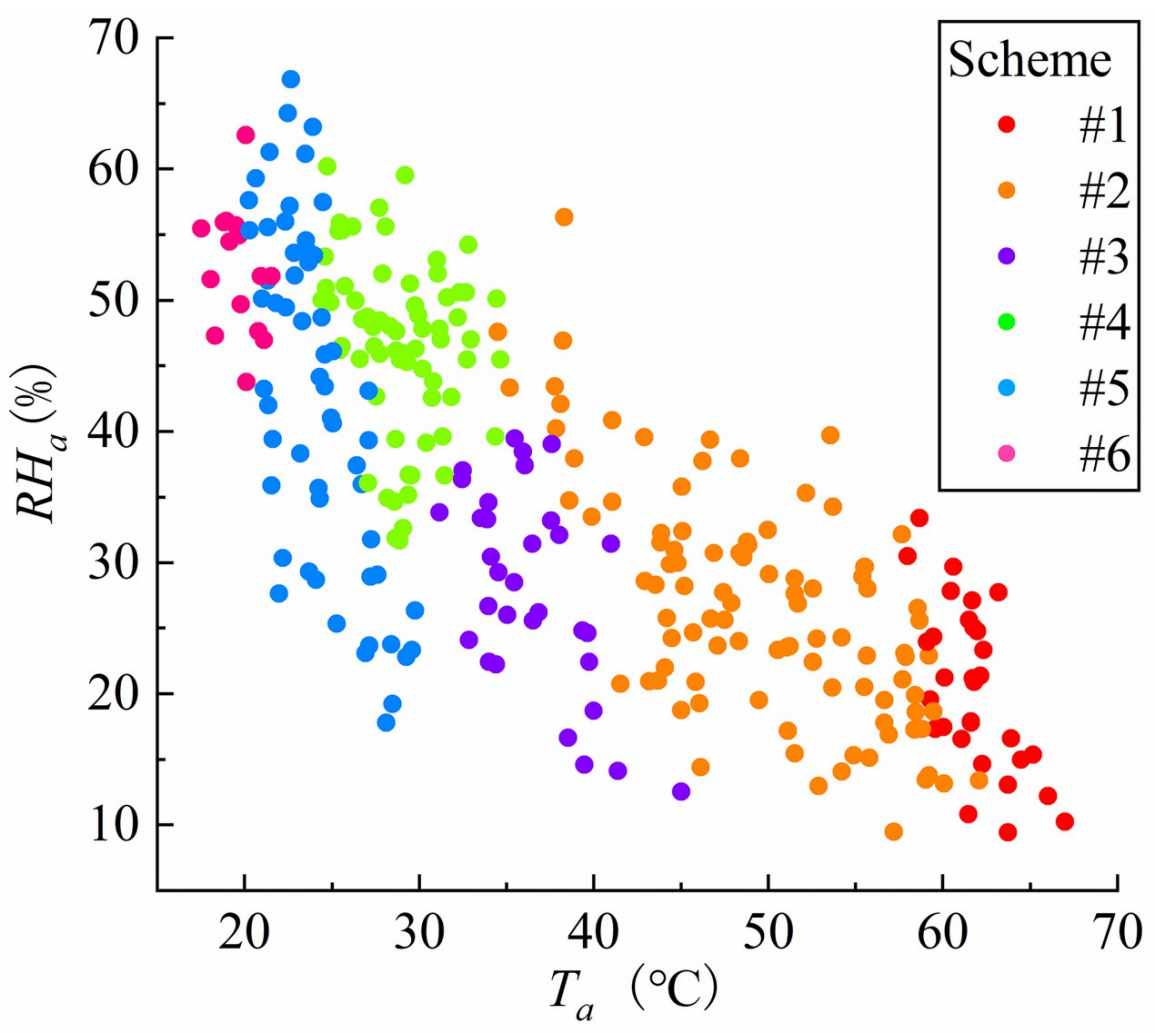
*T*_*ca*_ and schemes distribution of the sample point. The 300 samples selected consider the uniformity and representativeness of the sample points in the thermal environment state.

To train the classification model, a training set is first generated from the experimental data in Section 3. To avoid the sample points selection aimlessly, the optimal Latin hypercube experimental design method was applied to obtain 300 samples as the training set, as shown in Fig 13.

The models were trained in toolboxes of MATLAB. To avoid over-fitting, k-fold Cross Validation was set to 10 [30]. The *t*, *T*_*ca*_, *T*_*atm*_, *T*_*s*_, *T*_*w*_, *RH*_*a*_, *RH*_*atm*_, and PMV were used as the classification input labels, and the output was the serial number of the level.

**Fig 14:**
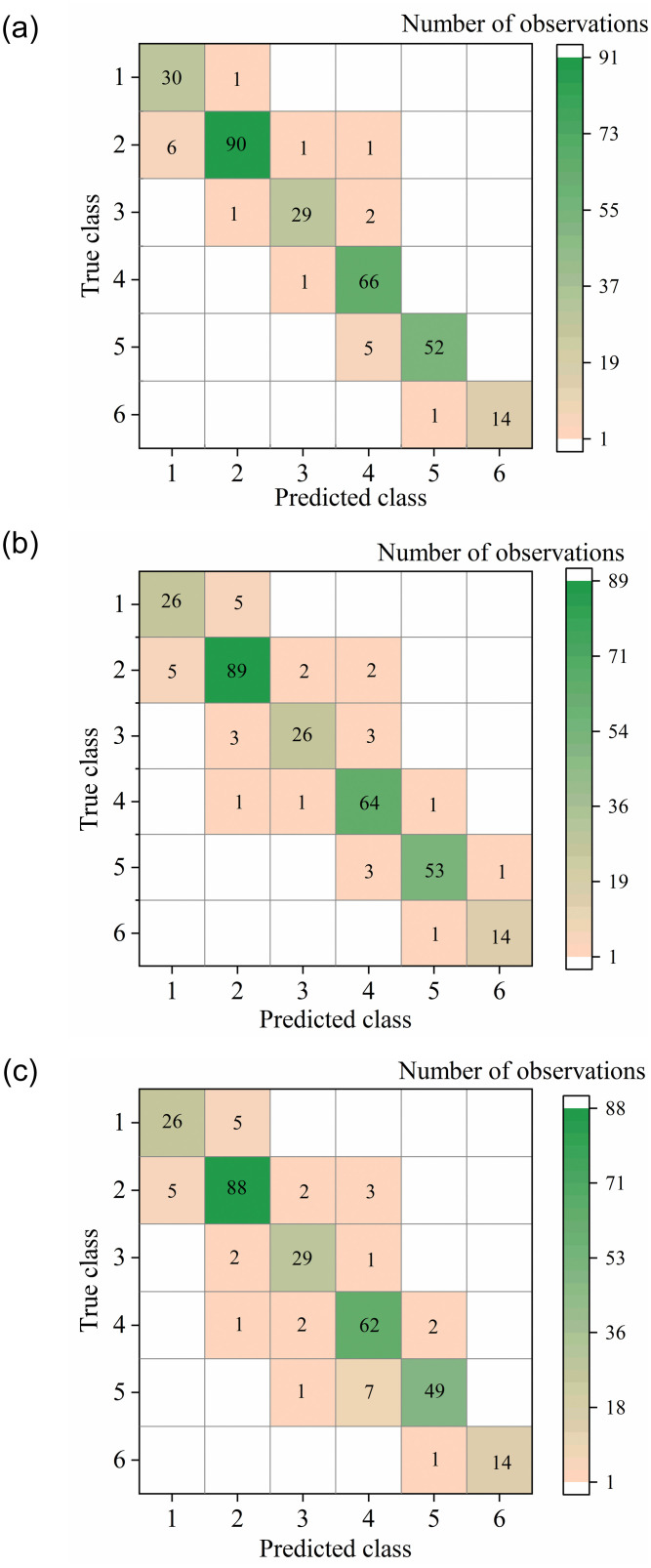
Confusion matrix for classification models training. Classification mistakes mainly happen between scheme 1 and scheme 2 and between scheme 4 and scheme 5. (a): Cubic SVM. (b): RF. (c): Weighted KNN.

The accuracy is used to evaluate the performance of the classification models in Table 4. The confusion matrix for classification in Fig 14 shows that classification mistakes mainly happen between level 1 and 2 and between level 4 and 5. To test the classification models, 62 groups of test data are used and the test results are shown in Table 4. Compared with the analysis of the above classification models, the SVM using cubic kernel function has the highest accuracy in training, while it also has well accuracy in testing. Therefore, the study applies the trained Cubic SVM model to identify the cabin thermal environment level.

**Table 4:** Accuracy of different classification models.

Case	SVM			Decision Tree			RF	Weighted KNN
	Quadratic	Cubic	Gaussian Kernel scale = 2.6	Split = 4	Split = 20	Split = 100	Learners = 30	Neighbors = 10
Training	93.0%	93.7%	92.3%	77.0%	87.7%	87.7%	90.7%	89.3%
Testing	93.5%	95.2%	91.9%	82.3%	93.5%	93.5%	93.5%	90.3%

Synthesis of the above analysis process, a complete cabin pre-conditioning strategy decision model is established. Firstly, measure and predict the cabin environment variables with the weather data inputted; then input the predicted variables and weather data to Cubic SVM to predict its climate level category and match the required scheme.

### Case studies for cabin pre-conditioning

Case studies are conducted to verify the effect of the pre-conditioning strategies matching by the Cubic SVM model. The case studies are chosen and analyzed in Table 5. The analysis results show that when the environmental characteristics are between the boundaries of the environment levels, matching scenarios and solutions are probably inconsistent with the actual optimal solution; otherwise, the model can effectively give the ideal solution. All the case studies keep thermal comfort index *PMV* within a suitable range, which is the most concerning element to passengers.

**Table 5:** Case study for pre-conditioning analysis.

Case	*T*_*atm*_ (°C)	*RH*_*atm*_ (%)	*I* (W/m^2^)	*T*_*ca*_ (°C)	*RH*_*ca*_ (%)	*T*_*s*1_ (°C)	*T*_*s*2_ (°C)	*T*_*w*1_ (°C)	*T*_*w*2_ (°C)	*PMV* at 0 min	*PPD* at 0 min	Actual level	Predicted level	*PMV* at 10 min
1	40.09	25.93	938	65.17	15.38	69.07	61.14	75.02	61.92	3	100	1	1	0.78
2	34.95	26.21	571.9	45.7	24.66	44.02	41.19	40.77	41.07	3	100	2	2	0.24
3	30.7	22.67	42.83	39.73	22.41	39.58	38.66	39.51	40.17	2.35	89.61	3	3	0.54
4	34.33	56.59	0	34.51	47.59	34.58	34.35	33.89	34.8	2.04	78.58	4	4	0.88
5	25.03	77.4	0	23.46	61.16	23.55	23.33	23.23	23.7	0.95	24.13	5	5	0.51
6	18.26	78.34	17.75	15.92	53.06	16.07	15.85	15.74	16.1	0.04	5.03	6	6	0.36
7	28.62	58.04	126	37.57	39.04	38.04	36.74	37.83	37.34	2.29	87.98	2	3	0.73
8	27.49	60.58	201.6	33.16	41.87	33.38	32.2	33.88	32.48	1.85	69.39	3	4	0.75
9	27.72	65.95	0	24.6	53.35	24.67	24.55	24.19	24.94	1.03	27.48	5	4	0.52

**Fig 15:**
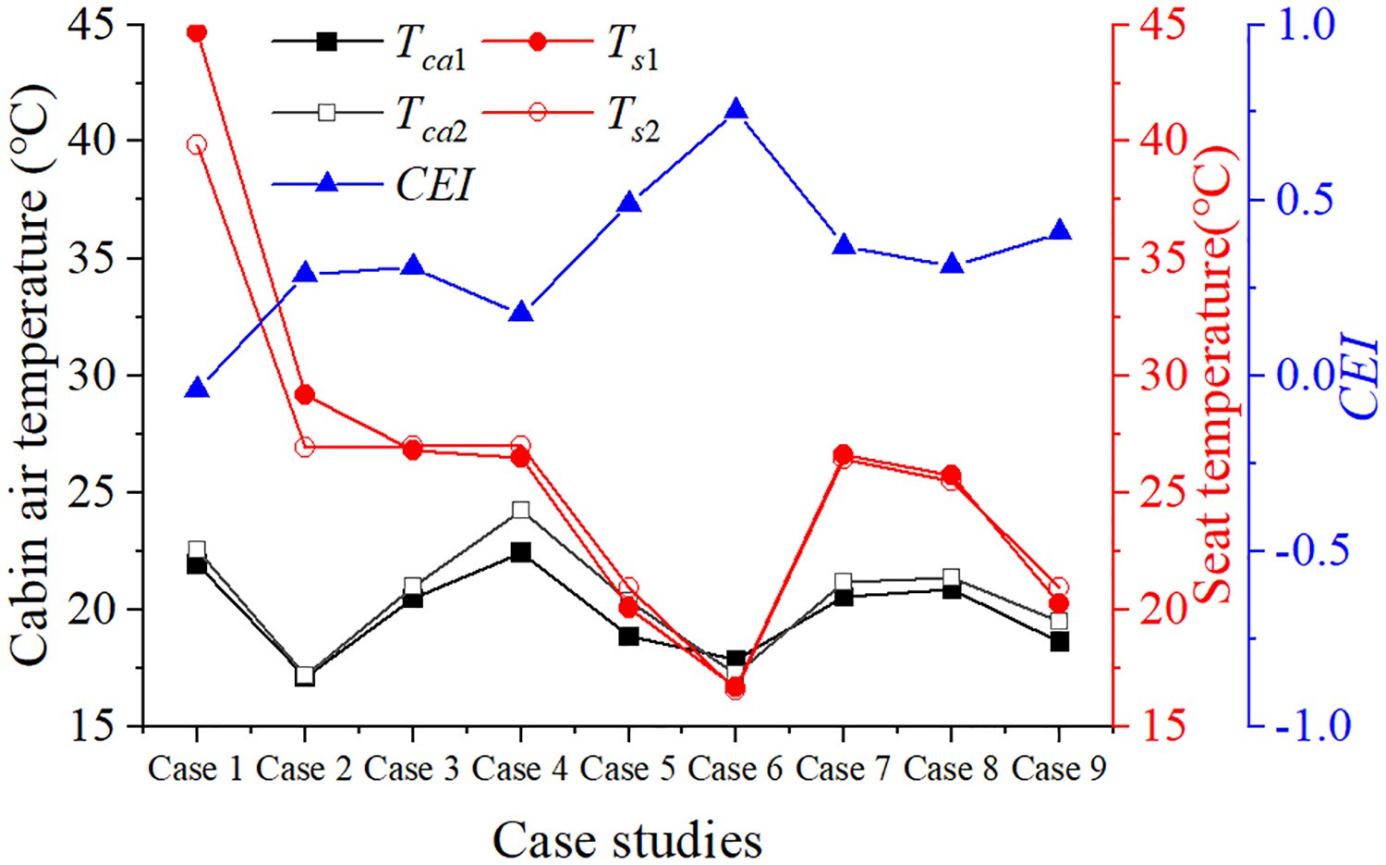
The pre-conditioning results for case study. All case studies achieve a *CEI* rating of 0 or higher, bringing cabin heat down to an acceptable value.

Cases 1 to 6 apply the corresponding ideal parameters schemes for cabin pre-conditioning, with the cabin air temperature changes shown in Fig 15. With the specified scheme predicted, the pre-conditioning system adjusts the cabin air temperature to a suitable temperature range within 10 minutes. The pre-conditioning parameters developed for different thermal environment levels meet the thermal comfort needs of passengers entering the cabin. For Case 7 to Case 9, although the decision scheme of these cases is inconsistent with the ideal, the pre-conditioning effect is also comfortable and satisfactory under the solution provided by the model.

According to the above analysis results, when the parked vehicle applies the cabin pre-conditioning scheme according to its scenario level, it can be expected to maximize the overall benefits to effectively improve the thermal comfort.

## Conclusion

The thermal states of the vehicle cabin fail to meet the human comfort objectives under the environmental conditions like solar radiation during parking. Based on passenger multi-target satisfaction needs, the study has developed a data-driven decision model to determine cabin pre-conditioning solutions efficiently and satisfactorily at different environmental conditions. In addition, the model quantifies passenger satisfaction considering comprehensive factors during cabin pre-conditioning. Notably, the following work is conducted:

1. The research rates the thermal states levels for cabin pre-conditioning considering diversity of thermal environmental conditions. A data-driven decision model with the Cubic SVM algorithm was established to identify the thermal environment levels and match satisfactory pre-conditioning solutions.
2. The characteristics of cabin thermal environment are analyzed from experiments in tropical desert climate. With the model for cabin thermal environment and reliable data on cabin thermal characteristics, the thermal responses on cabin air and structural under the effects of climate conditions and HVAC airflow is analyzed.
3. Considering the multiple satisfaction objectives, a comprehensive evaluation index *CEI* model is proposed to evaluate passengers’ satisfaction in pre-conditioning on both thermal comfort, seat temperature, air quality, and energy consumption. With the assessments for passengers’ satisfaction in pre-conditioning, cabin pre-conditioning strategies are designed to different environment levels based on *CEI*.
4. The performance of different temperatures, air volume and internal circulation ratios were compared for cabin pre-conditioning. The high cabin temperature increases the proportional demand for internal circulation, and the high ventilation speed shows high efficiency and energy efficiency.
5. The decision algorithm of SVM, Decision Tree, and KNN are compared in environment levels identification. The Cubic SVM algorithm shows high effectiveness in identify thermal environment levels with 92.3% accuracy. Several scene cases are carried out and both thermal environments are improved within multi-satisfaction objectives.

## Supporting information

S1 Appendix Equations derivation process. (PDF)

S2 Appendix Simulation results analysis. (PDF)
