# Supplementary material for: Environmental conditions driven method for automobile cabin pre-conditioning with multi-satisfaction objectives
Source: PLoS One. 2022 May 23;17(5):e0266672. doi: 10.1371/journal.pone.0266672 (PMC9126406; doi:10.1371/journal.pone.0266672)
Supplement: S1 Appendix — (PDF) [file pone.0266672.s001.pdf]

S1 Appendix. Equations derivation process.

The air and walls in the passenger compartment of a car follow the conservation of energy and mass during heat transfer, which leads to Equation 8-13.

(1) The process of deriving Eq (8)

According to the conservation of energy, the change of heat in the zone 1 of cabin air is equal to the difference between the heat input and the heat output,

$$\Delta Q_{cabin1} = \Delta Q_{input1} - \Delta Q_{\mathrm{output1}} \tag{16}$$

According to the definition, the heat of air is given by the change in mass, specific heat capacity and temperature as,

$$\Delta Q_{cabin1} = c_{ca} m_{ca1} \frac{\mathrm{d}T_{ca1}}{\mathrm{d}t} \tag{17}$$

The heat input to the vehicle cabin consists of the ambient environment input ΔQ_{ca1} and the supplied air for HVAC ΔQ_{ac1}, where the supplied air exchanges heat with the air in zone 1 so $\Delta Q_{ac1} = \delta \Delta m_{ac1} c_{ca} \left(T_{ac1} - T_{ca1}\right)$. Therefore, the heat input can be expressed as,

$$\Delta Q_{input1} = \Delta Q_{ca1} + \Delta Q_{ac1} = \Delta Q_{ca1} + \delta \Delta m_{ac1} c_{ca} \left(T_{ac1} - T_{ca1}\right) \tag{18}$$

where ambient environment input is mainly from solar radiation and convective heat transfer,

$$\Delta Q_{ca1} = \Delta Q_{solar-w1} + \Delta Q_{solar-s1} + \Delta Q_{w1-s1} + \Delta Q_{s1-w1} + \Delta Q_{ca1-w1} + \Delta Q_{ca1-s1} \tag{19}$$

The heat output of the air in zone 1 is mainly carried out with the heat exchange in zone 2,

$$\Delta Q_{\mathrm{output1}} = \Delta m_{ca1-ca2} c_{ca} \left(T_{ca1} - T_{ca2}\right) \tag{20}$$

Substituting Eqs (17), (18) and (20) into Eq(16),

$$c_{ca} m_{ca1} \frac{\mathrm{d}T_{ca1}}{\mathrm{d}t} = \Delta Q_{ca1} + \delta \Delta m_{ac1} c_{ca} \left(T_{ac1} - T_{ca1}\right) - \Delta m_{ca1-ca2} c_{ca} \left(T_{ca1} - T_{ca2}\right) \tag{21}$$

Eq(8) is obtained by converting the Eq(21).

(2) The process of deriving Eq (9)

According to the conservation of energy, the change of heat in the zone 2 of cabin air is equal to the difference between the heat input and the heat output,

$$\Delta Q_{cabin2} = \Delta Q_{input2} - \Delta Q_{\mathrm{output2}} \tag{22}$$

According to the definition, the heat of air is given by the change in mass, specific heat capacity and temperature as,

$$\Delta Q_{cabin2} = c_{ca} m_{ca2} \frac{\mathrm{d}T_{ca2}}{\mathrm{d}t} \tag{23}$$

The heat input to the vehicle cabin consists of the ambient environment input ΔQ_{ca2} and the supplied air for HVAC ΔQ_{ac2}, where the supplied air from the front vents and the rear vents

exchanges heat with the air in zone 2 so $\Delta Q_{ac2} = (1-\delta)\Delta m_{ac1} c_{ca}(T_{ac1} - T_{ca2}) + \Delta m_{ac2} c_{ca}(T_{ac2} - T_{ca2})$.

Therefore, the heat input can be expressed as,

$$\Delta Q_{input2} = \Delta Q_{ca2} + (1-\delta)\Delta m_{ac1} c_{ca}(T_{ac1} - T_{ca2}) + \Delta m_{ac2} c_{ca}(T_{ac2} - T_{ca2}) \quad (24)$$

where ambient environment input is mainly from solar radiation and convective heat transfer,

$$\Delta Q_{ca2} = \Delta Q_{solar-w2} + \Delta Q_{solar-s2} + \Delta Q_{w2-s2} + \Delta Q_{s2-w2} + \Delta Q_{ca2-w2} + \Delta Q_{ca2-s2} \quad (25)$$

The heat output of the air in zone 1 is mainly carried out with the heat exchange in zone 2,

$$\Delta Q_{\mathrm{output1}} = \Delta m_{ca1-ca2} c_{ca}(T_{ca1} - T_{ca2}) \quad (26)$$

Substituting Eqs (23), (24) and (26) into Eq(22),

$$c_{ca} m_{ca2} \frac{\mathrm{d}T_{ca2}}{\mathrm{d}t} = \Delta Q_{ca2} + (1-\delta)\Delta m_{ac1} c_{ca}(T_{ac1} - T_{ca2}) + \Delta m_{ac2} c_{ca}(T_{ac2} - T_{ca2}) + \Delta m_{ca1-ca2} c_{ca}(T_{ca1} - T_{ca2}) \quad (27)$$

Eq(9) is obtained by converting the Eq(22).

(3) The process of deriving Eq (10)

According to the conservation of energy, the change of heat in the zone 1 of seats is equal to the difference between the heat input and the heat output,

$$\Delta Q_{s1} = \Delta Q_{si1} - \Delta Q_{so1} \quad (28)$$

According to the definition, the heat of seat is given by the change in mass, specific heat capacity and temperature as,

$$\Delta Q_{s1} = c_s m_{s1} \frac{\mathrm{d}T_{s1}}{\mathrm{d}t} \quad (29)$$

Heat input to the seat comes from external radiation, is expressed as,

$$\Delta Q_{si1} = \Delta Q_{solar-s1} + \Delta Q_{ca1-s1} \quad (30)$$

Seats dissipate heat by radiating outwards,

$$\Delta Q_{so1} = \Delta Q_{s1-w1} + \Delta Q_{s1-w2} \quad (31)$$

Substituting Eqs (29), (30) and (31) into Eq(28),

$$c_s m_{s1} \frac{\mathrm{d}T_{s1}}{\mathrm{d}t} = \Delta Q_{solar-s1} + \Delta Q_{ca1-s1} - \Delta Q_{s1-w1} - \Delta Q_{s1-w2} \quad (32)$$

Eq(10) is obtained by converting the Eq(32).

(4) The process of deriving Eq (11)

According to the conservation of energy, the change of heat in the zone 1 of wall is equal to the difference between the heat input and the heat output,

$$\Delta Q_{s1} = \Delta Q_{si1} - \Delta Q_{so1} \quad (33)$$

According to the definition, the heat of seat is given by the change in mass, specific heat capacity and temperature as,

$$\Delta Q_{w1} = c_w m_{w1} \frac{\mathrm{d}T_{w1}}{\mathrm{d}t} \quad (34)$$

Heat input to the seat comes from external radiation, is expressed as,

$$\Delta Q_{wi1} = \Delta Q_{solar-w1} + \Delta Q_{ca1-w1} \quad (35)$$

Seats dissipate heat by radiating outwards,

$$\Delta Q_{wo1} = \Delta Q_{w1-s1} + \Delta Q_{w1-s2} + \Delta Q_{w1-sky} \tag{36}$$

Substituting Eqs (34), (35) and (36) into Eq(33),

$$\begin{aligned} c_w m_{w1} \frac{\mathrm{d}T_{w1}}{\mathrm{d}t} &= \Delta Q_{solar-w1} + \Delta Q_{ca1-w1} + \Delta Q_{atm-w1} + \Delta Q_{w2-w1} \\ &- \Delta Q_{w1-s1} - \Delta Q_{w1-s2} - \Delta Q_{w1-sky} \end{aligned} \tag{37}$$

Eq(11) is obtained by converting the Eq(37).

(5) The process of deriving Eq (12)

According to the conservation of mass, the change of air moisture mass in the cabin in the zone 1 is equal to the difference between the moisture input and the moisture output,

$$\Delta m_{w1} = m_{ca1} \frac{\mathrm{d}w_{ca1}}{\mathrm{d}t} = \Delta m_{wi1} - \Delta m_{wo1} \tag{38}$$

The moisture mass in the cabin is affected by the water vapour content from HVAC. The water vapour mass from HVAC can be expressed as,

$$\Delta m_{wi1} = \delta \Delta m_{ac1} \left(w_{ac1} - w_{ca1} \right) \tag{39}$$

Air exchange between zones 1 and 2 causes a change in water vapour mass in zone 1,

$$\Delta m_{wo1} = \Delta m_{ca1-ca2} \left(w_{ca1} - w_{ca2} \right) \tag{40}$$

Substituting Eqs (39) and (40) into Eq(38),

$$m_{ca1} \frac{\mathrm{d}w_{ca1}}{\mathrm{d}t} = \delta \Delta m_{ac1} \left(w_{ac1} - w_{ca1} \right) - \Delta m_{ca1-ca2} \left(w_{ca1} - w_{ca2} \right) \tag{41}$$

Eq(12) is obtained by converting the Eq(41).

(6) The process of deriving Eq (12)

According to the conservation of mass, the change of air moisture mass in the cabin in the zone 1 is equal to the difference between the moisture input and the moisture output,

$$\Delta m_{w2} = m_{ca2} \frac{\mathrm{d}w_{ca2}}{\mathrm{d}t} = \Delta m_{wi2} - \Delta m_{wo2} \tag{42}$$

The moisture mass in the zone 2 is affected by the water vapour content from HVAC in front vent and rear vent,

$$\Delta m_{wi1} = \left(1 - \delta\right) \Delta m_{ac1} \left(w_{ac1} - w_{ca2} \right) + \Delta m_{ac2} \left(w_{ac2} - w_{ca2} \right) \tag{43}$$

Air exchange between zones 1 and 2 causes a change in water vapour mass in zone 1,

$$\Delta m_{wo2} = \Delta m_{ca1-ca2} \left(w_{ca2} - w_{ca1} \right) \tag{44}$$

Substituting Eqs (43) and (44) into Eq(42),

$$\begin{aligned} m_{ca1} \frac{\mathrm{d}w_{ca1}}{\mathrm{d}t} &= \left(1 - \delta\right) \Delta m_{ac1} \left(w_{ac1} - w_{ca2} \right) + \Delta m_{ac2} \left(w_{ac2} - w_{ca2} \right) \\ &+ \Delta m_{ca1-ca2} \left(w_{ca1} - w_{ca2} \right) \end{aligned} \tag{45}$$

Eq(12) is obtained by converting the Eq(45).
