# Supplementary material for: Environmental conditions driven method for automobile cabin pre-conditioning with multi-satisfaction objectives
Source: PLoS One. 2022 May 23;17(5):e0266672. doi: 10.1371/journal.pone.0266672 (PMC9126406; doi:10.1371/journal.pone.0266672)
Supplement: S2 Appendix — (PDF) [file pone.0266672.s002.pdf]

S2 Appendix. Simulation results analysis.

The simulation results of parts of scenarios are shown in Appendix Table A1.

Table A1 Analysis results of main parameter combinations.

Scheme order	[T_R (°C), v_{ac} (m/s), β (%)]	T_{ca1} (°C)	T_{ca2} (°C)	T_{s1} (°C)	T_{s2} (°C)	PMV_1	PDD_1	CEI
1	[6, 7.5, 100]	23.851	22.625	46.890	41.237	0.923	22.976	-0.099
	[6, 7.5, 75]	27.013	26.026	48.560	43.087	1.195	35.011	-0.202
	[6, 7.5, 50]	28.934	27.242	49.440	43.998	1.361	43.435	-0.263
	[6, 7.5, 0.25]	30.243	28.847	50.222	44.878	1.470	49.431	-0.295
	[6, 7.5, 0]	31.185	31.131	50.996	45.967	1.550	53.810	-0.309
	[8, 7.5, 100]	25.995	29.731	49.187	45.302	1.108	30.889	-0.189
	[10, 7.5, 100]	44.672	45.597	59.707	56.626	2.895	98.550	-0.976
2	[6, 7.5, 100]	17.120	18.627	34.025	30.998	0.232	6.125	0.127
	[6, 7.5, 0]	27.927	27.986	39.847	36.868	1.521	52.014	-0.272
	[6, 4.5, 100]	20.686	22.138	36.004	33.674	0.62	13.05	0.071
	[6, 4.5, 0]	27.882	31.590	40.747	38.585	1.493	50.546	-0.268
	[8, 7.5, 100]	18.762	20.221	34.964	32.011	0.442	9.081	0.191
	[8, 7.5, 75]	24.719	25.615	38.111	35.355	1.158	33.237	-0.076
	[8, 4.5, 100]	22.448	23.257	36.885	34.582	0.827	19.426	0.060
	[8, 4.5, 75]	27.145	27.325	39.561	36.854	1.347	42.720	-0.199
	[8, 1.5, 100]	34.186	34.841	44.118	42.234	1.899	71.973	-0.593
3	[6, 7.5, 100]	13.219	13.261	21.925	21.437	-0.300	6.920	0.235
	[6, 7.5, 0]	22.574	22.643	27.574	27.573	0.710	15.870	0.258
	[8, 7.5, 100]	14.376	14.933	22.749	22.531	-0.172	5.610	0.299
	[8, 7.4, 0]	22.991	24.306	27.987	28.283	0.754	16.986	0.312
	[10, 7.4, 100]	16.364	16.485	24.079	23.698	0.061	5.076	0.330
	[10, 7.4, 75]	18.983	19.425	25.441	25.467	0.349	7.532	0.352
	[12, 7.4, 100]	17.859	18.020	24.741	24.872	0.226	6.063	0.350
	[12, 7.4, 75]	20.165	21.039	26.263	26.419	0.475	9.7178	0.372
4	[14, 7.4, 75]	23.677	23.716	26.349	26.155	1.134	32.099	0.136
	[14, 4.5, 100]	23.410	23.531	26.344	26.069	1.082	29.716	0.167
	[14, 4.5, 75]	18.773	19.509	23.542	23.635	0.490	10.026	0.385
	[14, 1.5, 100]	23.139	23.388	26.451	26.367	0.956	24.292	0.193
	[14, 1.5, 75]	25.336	25.572	27.715	27.540	1.249	37.693	0.062
5	[14, 7.4, 0]	21.479	22.737	22.863	23.357	0.910	22.487	0.392
	[14, 4.5, 0]	21.675	22.095	22.819	23.208	0.916	22.715	0.393
	[14, 4.5, 75]	20.018	20.062	21.842	21.883	0.685	14.874	0.383
	[14, 4.5, 50]	20.579	21.550	22.242	22.774	0.775	17.655	0.382
	[14, 4.5, 25]	20.844	22.325	22.577	23.097	0.819	19.158	0.399
	[14, 1.5, 100]	19.397	20.240	21.688	22.228	0.546	11.243	0.390

	[14, 1.5, 75]	20.063	22.543	22.512	23.126	0.689	14.967	0.375
	[14, 1.5, 0]	22.241	22.667	23.280	23.742	0.967	24.772	0.366
6	[14, 4.5, 0]	14.680	14.624	14.057	14.352	-0.117	5.285	0.571
	[14, 1.5, 100]	11.647	14.622	12.934	13.817	-0.480	9.801	0.4238
	[14, 1.5, 75]	13.349	13.317	13.320	13.637	-0.267	6.5135	0.461
	[14, 1.5, 0]	15.495	12.176	13.797	13.754	-0.025	5.013	0.574
	[-, 7.4, 75]	14.780	14.756	14.241	14.505	-0.104	5.222	0.581
	[-, 7.4, 0]	16.685	16.070	15.394	15.228	0.109	5.247	0.694
